# GTestimate: improving relative gene expression estimation in scRNA-seq using the Good–Turing estimator

**DOI:** 10.1093/gigascience/giaf084

**Published:** 2025-10-08

**Authors:** Martin Fahrenberger, Christopher Esk, Jürgen A Knoblich, Arndt von Haeseler

**Affiliations:** Center for Integrative Bioinformatics Vienna (CIBIV), Max Perutz Labs, University of Vienna and Medical University of Vienna, Vienna BioCenter (VBC), 1030 Vienna, Austria; Vienna Biocenter PhD Program, a Doctoral School of the University of Vienna and the Medical University of Vienna, 1030 Vienna, Austria; Institute of Molecular Biology, University of Innsbruck, 6020 Innsbruck, Austria; Institute of Molecular Biotechnology of the Austrian Academy of Science (IMBA), Vienna BioCenter (VBC), 1030 Vienna, Austria; Institute of Molecular Biotechnology of the Austrian Academy of Science (IMBA), Vienna BioCenter (VBC), 1030 Vienna, Austria; Department of Neurology, Medical University of Vienna, 1090 Vienna, Austria; Ludwig Boltzmann Institute for Network Medicine, University of Vienna, 1090 Vienna, Austria

**Keywords:** scRNA-seq, normalization, gene expression, Good–Turing estimator, deep sequencing, targeted amplification

## Abstract

**Background:**

Single-cell RNA-seq suffers from unwanted technical variation between cells, caused by its complex experiments and shallow sequencing depths. Many conventional normalization methods try to remove this variation by calculating the relative gene expression per cell. However, their choice of the maximum likelihood estimator is not ideal for this application.

**Results:**

We present *GTestimate*, a new normalization method based on the Good–Turing estimator, which improves upon conventional normalization methods by accounting for unobserved genes. To validate *GTestimate*, we developed a novel cell-targeted PCR amplification approach (cta-seq), which enables ultra-deep sequencing of single cells. Based on these data, we show that the Good–Turing estimator improves relative gene expression estimation and cell–cell distance estimation. Finally, we use *GTestimate*’s compatibility with Seurat workflows to explore 4 example datasets and show how it can improve downstream results.

**Conclusion:**

By choosing a more suitable estimator for the relative gene expression per cell, we were able to improve scRNA-seq normalization, with potentially large implications for downstream results. *GTestimate* is available as an easy-to-use R-package and compatible with a variety of workflows, which should enable widespread adoption.

## Introduction

**Figure 1:**
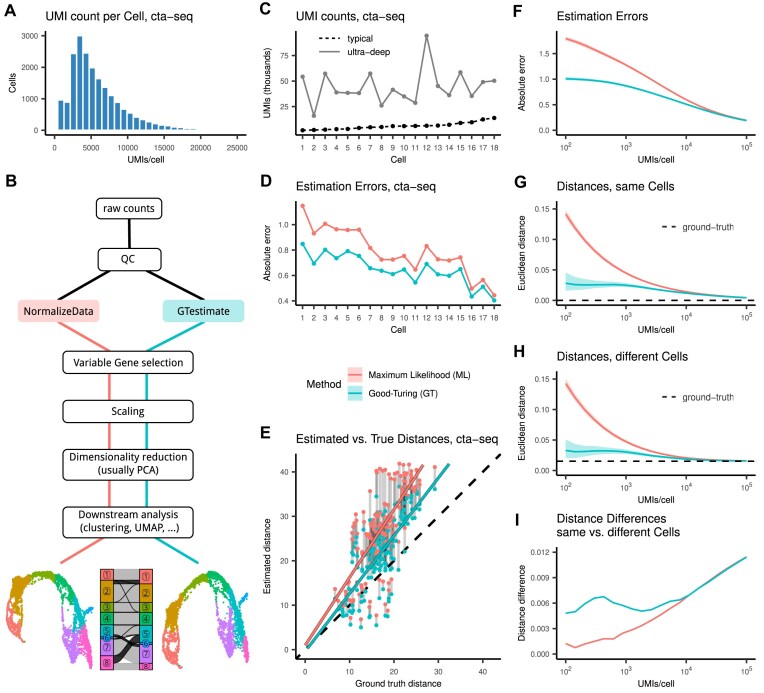
(A) Histogram of *UMIs/cell* for 17,653 cells in the cta-seq experiment before amplification. (B) Schema of a scRNA-seq analysis showing where *GTestimate* integrates into the workflow. (C) *UMIs/cell* for the 18 selected cells in the cta-seq experiment, before (*typical*) and after (*ultra-deep*) amplification. Cells ordered based on *UMIs/cell* in the *typical* cta-seq data. (D) Absolute error of the relative gene expression estimation in the cta-seq experiment. (E) Euclidean cell–cell distances in PCA space in the cta-seq experiment. (F) Average absolute estimation error of the relative gene expression of a cell when subsampled to different *UMIs/cell*. (G, H) Mean Euclidean cell–cell distance in relative gene expression space, between 2 independent random samples of the same cell (G) between independent random samples of 2 different cells (H). (I) Difference between the mean cell–cell distances in (G) and (H). Colored ribbons in (F), (G), and (H) represent the $5\%-95\%$ quantile range.

Single-cell RNA-seq (scRNA-seq) provides new insights into cell diversity, differentiation, and disease [1–3]. These insights are enabled by affordable high-throughput methods for the parallel sequencing of thousands of cells [4, 5]. However, they require many experimental steps, whose efficiency differs between cells, leading to high variability in the number of mRNAs captured. Additionally, sequencing depths as low as 20,000 reads per cell [6] and the nature of parallel sequencing introduce stochastic variation [5, 7, 8]. After accounting for PCR duplicates among reads, a median of $\sim$5,000 *UMIs/cell* (number of sequenced mRNA molecules per cell) with a range of $\sim$500 to 20,000 *UMIs/cell* is typical for a high-quality sample (Fig. 1A). This high technical variation between cells results in a low signal-to-noise ratio, which makes data analysis challenging.

During data processing (Fig. 1B), *global-scaling normalization* methods [8] such as Seurat’s *NormalizeData* [9], scran’s *computeSumFactors* [10, 11], or scanpy’s *normalize_total* [12] account for the variation in *UMIs/cell* by calculating a single scaling factor (or size factor) per cell. Despite its simplicity, this approach has been shown to outperform more complex methods [13].

*Global-scaling normalization* inherently requires the calculation of the relative gene expression levels per cell. Although not typically discussed as such, the calculation used by these methods is a maximum likelihood (ML) estimation [14] of the relative gene expression frequency per cell.


(ML) \begin{eqnarray*}
\widehat{f_g}^{ML} = \frac{c_g}{\sum _i c_i}
\end{eqnarray*}

where $c$ denotes the transcriptomic profile of the cell with a count $c_g$ for each gene $g$.

However, at $\sim$5,000 *UMIs/cell*, only $\sim$2.5% of the $\sim$200,000 mRNA transcripts in a typical mammalian cell [15] are sequenced and many expressed genes remain unobserved, as evident by the low *genes/cell* observed in scRNA-seq experiments (Supplementary Fig. S1). ML then estimates the relative expression of unobserved genes as zero. This inherently leads to overestimation of the relative expression for observed genes, since the sum of all relative frequencies equals 1 ($\sum _g \widehat{f_g}^{ML} = 1$).

To reduce this overestimation, we propose a Simple Good–Turing (GT) estimator [16, 17].


(GT) \begin{eqnarray*}
\widehat{f_g}^{GT} = \left\lbrace \begin{array}{@{}l@{\quad }l@{}}\frac{(c_g+1)}{\sum _ic_i} \cdot \frac{S(N_{{c_g}+1})}{S(N_{c_g})}, & \text{for } c_g > 0\\
\hfil 0, & \text{for } c_g = 0 \end{array}\right.
\end{eqnarray*}

where $N_{c_g}$ denotes the number of genes with count $c_g$ in the cell, and $S()$ is a smoothing function following Gale and Sampson [17].

GT adjusts the relative expression estimates of observed genes, particularly those with low counts, based on the frequency of each count value in the cell. This even enables an estimate for the relative expression of unobserved genes (for further details, see Supplementary Materials 1.1).

In this study, we first compare the performance of GT and ML on novel ultra-deep sequencing data and then show how GT improves downstream results, by integrating it into standard scRNA-seq analysis workflows. To achieve this, we developed *GTestimate*, a new scRNA-seq normalization method centered on GT. *GTestimate* is an easy-to-use R-package designed to seamlessly replace Seurat’s *NormalizeData*.

## Results

### ultra-deep sequencing of single cells

Comparison between GT and ML requires ground-truth transcriptomic profiles of single cells. However, current simulation software cannot adequately emulate the complexity of scRNA-seq data, and the choice of simulator may affect benchmarking results [18]. We therefore designed a cell-targeted PCR amplification strategy (cta-seq), which enabled us to sequence a small set of selected cells, from a *typical* sequencing run, a second time at an *ultra-deep* sequencing depth. This *ultra-deep* sequencing data contain an average of 23 million reads (44,511 *UMIs*, 7,403 *genes*) per cell, a stark contrast to the average 16,965 reads (6,048 *UMIs*, 2,246 *genes*) for the same cells in the *typical* data (Supplementary Fig. S2). This represents a $\sim$7.4-fold increase in *UMIs/cell* (Fig. 1C) and a $\sim$3.3-fold increase in *genes/cell* (Supplementary Fig. S3). We then used the relative gene expression levels of these *ultra-deep* profiles as the ground truth for these cells.

### Performance of GT and ML

Based on the cta-seq data, we then evaluated GT and ML. When we applied GT and ML to the *typical* profiles and compared the results to the ground truth, GT consistently showed a lower estimation error across all 18 cells, by $\sim$17% on average (Fig. 1D).

Relative gene expression profiles are the basis of most scRNA-seq analysis (Fig. 1B), such as the calculation of cell–cell distances in principal component analysis (PCA) space (often used as a measure for the similarity between 2 cells). We therefore also calculated cell–cell distances between the *typical* profiles, once based on GT and once based on ML, and compared the results to the cell–cell distances between the *ultra-deep* profiles. We observed a 36% reduction of the distance estimation error when using GT instead of ML (Fig. 1E, Supplementary Table S1).

Since *UMIs/cell* vary drastically (Fig. 1A), we further assessed the performance of GT and ML at different *UMIs/cell*. We applied GT and ML to random subsamples of the cell with the highest *UMIs/cell* in the *ultra-deep* cta-seq data (cell 12, at 94,440 UMIs) and compared the estimates to the ground-truth expression profile of this cell. Similar to before (Fig. 1D), the estimation error for both GT and ML decreased with increasing *UMIs/cell*, and GT consistently showed a lower error than ML, especially at low *UMIs/cell* (Fig. 1F).

Next, we assessed the impact of *UMIs/cell* on cell–cell distances. We first compared the mean distance between 2 random samples of the same cell (cell 12), both sampled to the same *UMIs/cell*. This distance was calculated in relative gene expression space and should approach zero for high *UMIs/cell*. However, ML led to grossly overestimated distances at small *UMIs/cell* (Fig. 1G). The estimated distance after ML additionally showed strong correlation to the *UMIs/cell*, which is problematic as we assume that most of the observed variation in *UMIs/cell* is technical noise. In contrast, GT did not show correlation to the *UMIs/cell* and demonstrated lower distance estimation errors overall.

We then examined the distances between 2 distinct cells by also drawing random samples from the cell with the second highest *UMIs/cell* in the *ultra-deep* cta-seq data (cell 15, at 58,589 UMIs), which is of a different cell type. We calculated the distances between the sampled profiles of cell 12 and cell 15 at varying *UMIs/cell*. We again saw large overestimation of the distances when using ML, while using GT strongly reduced this error. For high *UMIs/cell*, the estimated distances converged to the true distance of 0.015 (Fig. 1H).

When based on ML, the estimated distances between identical cells (Fig. 1G) and distinct cells (Fig. 1H) were almost the same for low *UMIs/cell*. This makes it very difficult to distinguish between cell types. However, when we used GT as the basis for these distances, we saw a much clearer separation between identical cells and cells of different cell types, for cells with $< 10,000$  *UMIs/cell* (Fig. 1I).

### *GTestimate*’s impact on downstream results

After showing GT’s advantages for relative gene expression estimation and cell–cell distance estimation, we examined how our GT-based normalization method *GTestimate* impacts downstream results. The difference between *GTestimate* and other *global-scaling normalization* methods is only in the estimator used; all other settings can be adjusted to be equivalent to scran’s *computeSumFactors* or scanpy’s *normalize_total*, for example. At default settings, *GTestimate* behaves identically to *NormalizeData*, including the same log-transformation. We therefore used *NormalizeData*, as a representative of ML-based *global-scaling normalizations*, for all following comparisons. However, we would expect similar results when comparing to other *global-scaling normalization* methods.

**Figure 2:**
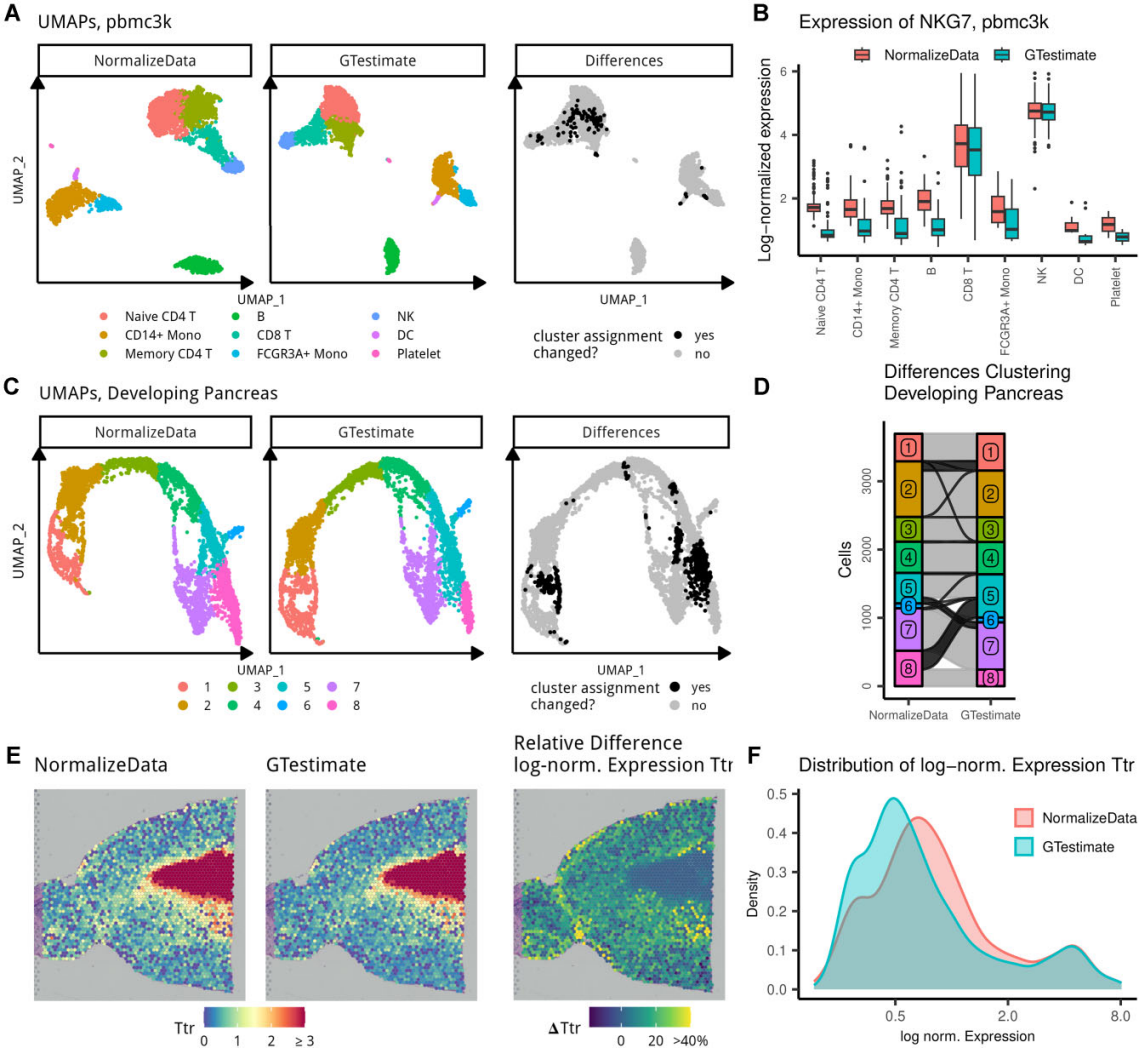
pbmc3k: (A) UMAPs based on *NormalizeData* and *GTestimate*, and UMAP highlighting differences in cluster assignment. (B) Boxplot showing log-normalized expression of *NKG7* per cell type (zeroes not shown). Developing Pancreas: (C) UMAPs based on *NormalizeData* and *GTestimate*, and UMAP highlighting differences in cluster assignment. (D) Sankey diagram showing the differences in cluster assignment based on *NormalizeData* and *GTestimate*. Spatial Transcriptomics: (E) log-normalized gene expression of *Ttr* based on *NormalizeData* and *GTestimate* as well as percent difference in log-normalized expression of *Ttr* between *NormalizeData* and *GTestimate*. (F) Density plot showing the distribution of log-normalized gene expression values of *Ttr* for *NormalizeData* and *GTestimate*.

Direct comparison of normalized gene expression values across different normalization methods (e.g., residual-based methods such as *SCTransform*) is often difficult due to varying scales and different data transformations. We therefore focus our initial comparison (Fig. 2) on *NormalizeData*, representing global-scaling normalization methods. This choice aligns with recent findings indicating that global-scaling methods (followed by a log-transformation with pseudo-count and PCA) typically match or outperform more complex approaches [13]. However, we also provide a downstream clustering-based comparison, including *SCTransform*.

We first assessed *GTestimate*’s impact on cell-type clustering by reanalyzing the pbmc3k dataset of peripheral blood mononuclear cells [19]. Here, normalization with *GTestimate* instead of *NormalizeData* resulted in 4.6% of cells being assigned to a different cluster (Fig. 2A), mostly among the naive CD4 T cells, memory CD4 T cells, and CD8 T cells.

We additionally analyzed a developing pancreas dataset [20], characterized by more gradual cell-type transitions compared to the pbmc3k dataset. After normalization with *GTestimate* instead of *NormalizeData*, 14.6% of cells were assigned to a different cluster (Fig. 2C, D).

While the correct classification of cells in both of these datasets remains unknown, our results in Fig. 1 suggest that *GTestimate* provides a better basis for this classification.

To examine the impact of *GTestimate* on the expression estimates of individual genes, we considered the log-normalized expression of cell-type specific marker genes in the pbmc3k dataset. As an example, we used *NKG7*, a highly specific NK-cell and *CD8*^+^ T-cell marker [21]. When using *GTestimate* instead of *NormalizeData*, the log-normalized expression of *NKG7* remained constant in NK cells and *CD8*^+^ T cells but was reduced in all other cell types (Fig. 2B). *GTestimate* therefore resulted in clearer separation of NK cells and *CD8*^+^ T cells from other cell types. We observed this for nearly all marker genes described in Seurat’s pbmc3k tutorial (Supplementary Fig. S4). These differences may explain some of the observed changes in clustering.

We also applied *GTestimate* to the spotwise normalization of a Spatial Transcriptomics dataset of the mouse brain [22]. In this dataset, normalization with *GTestimate* and *NormalizeData* resulted in 17 and 19 clusters, respectively (Supplementary Figs. S5, S6, S7); we therefore refrained from any cluster-based comparisons of *GTestimate* and *NormalizeData*. However, the spatial coordinates enabled examination of area-specific marker genes, independent of the clustering. As an example, we considered the log-normalized expression of the choroid plexus marker gene *Ttr* (Fig. 2E). When using *GTestimate*, we saw a reduction of the unspecific expression of *Ttr* for spots outside the choroid plexus. Here, *GTestimate* showed up to 50% reduction of the log-normalized expression, compared to *NormalizeData*, while expression estimates inside the choroid plexus remained constant (Fig. 2E). This resulted in clearer separation of the choroid plexus spots from the surrounding tissue, as shown by the distribution of expression values of *Ttr* (Fig. 2F).

When we additionally considered the *UMIs/spot* (Supplementary Fig. S8), we saw a negative correlation between the change in log-normalized expression of *Ttr* and *UMIs/spot*. This supports previous observations that *NormalizeData* overestimates the expression of *Ttr* in areas with low *UMIs/spot*. In contrast, *GTestimate* reduces this overestimation and improves the signal-to-noise ratio.

The datasets shown in Figs. 2A, C are widely used examples that highlight different aspects of scRNA-seq analysis. However, since these datasets lack ground-truth cell-type annotations, we cannot conclusively evaluate clustering accuracy based on them alone. Although the clear differences observed when using *GTestimate* instead of *NormalizeData*, together with our earlier results (Fig. 1), suggest improved relative gene-expression estimation with GT, this does not necessarily translate to better clustering performance. Direct benchmarking of clustering performance requires annotated data.

To address this, we analyzed a recently published PBMC scRNA-seq dataset from Fu et al. [23], which includes experimentally annotated cell types obtained via antibody-coated magnetic beads, providing a robust benchmark for clustering performance. We performed standard scRNA-seq analysis on this dataset, normalizing once with *GTestimate*, once with *NormalizeData*, and once with *SCTransform*, followed by unsupervised clustering.

Here we also included *SCTransform*, as clustering is a downstream analysis step where differences in scaling and transformations become intrinsic properties of each normalization method. Consequently, the effects of these differences should be interpreted as advantages or disadvantages inherent to each approach.

We assessed clustering performance by calculating the Adjusted Rand Index (ARI) between the unsupervised clustering results and the provided cell-type annotations. The ARI ranges from 0 to 1, with 0 indicating no agreement and 1 indicating perfect agreement between 2 clusterings. Because clustering outcomes strongly depend on the selected resolution parameter, we evaluated a broad range of resolutions from 0.1 to 1.5 (Supplementary Fig. S9).

Normalization with *GTestimate* produced higher ARI scores than *NormalizeData* at 14 of the 15 tested resolutions and outperformed *SCTransform* at 10 resolutions. Importantly, *GTestimate* also yielded the highest overall ARI (0.874), compared to 0.768 for *NormalizeData* and 0.822 for *SCTransform*. This superior maximum ARI is particularly relevant, as in practice, the clustering resolution is routinely adjusted to optimize results. By this criterion, normalization with *GTestimate* provides the best clustering accuracy for this dataset.

## Discussion

In summary, the estimation of relative gene expression is a central part of scRNA-seq data analysis, which has not received the same attention as other steps. We have shown that replacing the standard ML with GT improves relative gene expression estimation, without requiring expensive computations. By improving the signal-to-noise ratio at this basic level, our new normalization method *GTestimate* can have large impact on downstream results.

In the validation, we avoided potential issues with simulated data by employing a novel cell-targeted PCR amplification strategy to sequence the same cells at 2 vastly different *UMIs/cell*. This strategy may also be useful in other areas, such as the study of rare cell types. Additionally, the resulting dataset may serve as a benchmark for other methods.

*GTestimate* is available as an open-source R-package (https://www.github.com/Martin-Fahrenberger/GTestimate) and works with all common scRNA-seq data formats. While *GTestimate*’s default behavior is designed to seamlessly replace *NormalizeData*, it is also compatible with a wide variety of other workflows.

## Materials and Methods

### Implementation of *GTestimate*

The user-facing section of our *GTestimate* package was developed in R and handles input and output in the various supported data formats. The core implementation of the Simple Good–Turing estimator is written in C++ and is heavily based on Aaron Lun’s implementation for the edgeR R-package [24]. This core implementation includes the linear smoothing, which is necessary due to the sparsity of the frequencies of frequencies vector (i.e., the frequency of the count values). It further includes a rescaling step, which ensures that the estimated relative expression frequencies of all observed genes, plus the sum of probabilities of all unobserved genes (Supplementary Materials 1.1), add up to exactly 1 [17].

### cta-seq experiment

In the cta-seq experiment, we aimed to sequence a selected set of cells from a *typical* scRNA-seq library again at an *ultra-deep* sequencing depth. However, due to sequencing saturation, this quickly becomes prohibitively expensive. We therefore designed a PCR-based cell-targeted amplification strategy (cta-seq) to selectively amplify all transcripts from a small set of cells, through the use of primers specific to their cell barcode. This is similar to the TAP-seq protocol [25], which uses gene-specific primers to amplify all transcripts of certain genes.

#### Sequencing cta-seq, *typical*

To ensure high-quality input material, we used leftover cDNA from a previously sequenced sample [26], which had shown high *UMIs/cell* and *genes/cell*. The sample was taken out of −20°C storage and prepared for Illumina sequencing at the Vienna Biocenter Next Generation Sequencing facility using the 10X Dual Index Kit TT. We then split the resulting sequencing library into 2 aliquots and stored the second half again at −20°C. The first half was sequenced on a Illumina NovaSeq S4 in paired-end mode with a 2 × 150-bp read length and 400 million reads.

#### Sequencing cta-seq, *ultra-deep*

Based on the results from the *typical* sequencing run, we selected 18 cells of interest for the cta-seq experiment (see below). For these 18 cells, we designed PCR primers specific to their cell barcodes. We used the second aliquot of the previously prepared sequencing library and split it further into 18 individual reactions, one for each targeted cell. We then performed 3 rounds of PCR amplification with the respective primers using Amplitaq Gold 360 MM (ThermoFisher, cat.: 4398886) supplemented with EvaGreen dye (Biotium, cat.: 31000). We used the following programs in a total volume of 50 µL. PCR1: 1. 95°C, 10 min; 2. 62°C, 30 s; 3. 72°C, 2 min; 4. Return to 2, ×2; 5. 95°C, 25 s; 6. 62°C, 30 s; 7. 72°C, 2 min, fluorescence measurement; 8. 72°C, 15 s; 9. Return to 5, ×16. PCR2: 1. 95°C, 10 min; 2. 62°C, 30 s; 3. 72°C, 2 min; 4. Return to 2, ×2; 5. 95°C, 25 s; 6. 62°C, 30 s; 7. 72°C, 2 min, fluorescence measurement; 8. 72°C, 15 s; 9. Return to 5, ×16. PCR3: 1. 95°C, 10 min; 2. 67°C, 30 s; 3. 72°C, 2 min; 4. Return to 2, ×2; 5. 95°C, 25 s; 6. 67°C, 30 s; 7. 72°C, 2 min, fluorescence measurement; 8. 72°C, 15 s; 9. Return to 5, ×8. Reactions were stopped in step 8 according to fluorescent measurements in log phase. Reaction input in PCRs 2 and 3 were 0.5 µL of the previous reaction. Resulting reactions were purified and pooled for Illumina sequencing on a NovaSeq S4 in paired-end mode with a 2 × 150-bp read length and 400 million reads. The primer sequences used can be found in Supplementary Table S2, and PCR1 primers were designed with varying lengths to achieve similar melting temperatures.

### Data analysis

All data analysis was performed in R (v4.3.1) using Seurat (v5.0.0) functions at default settings unless stated otherwise.

#### Data analysis, cta-seq *typical* depth

We first processed the *typical* depth sequencing data using CellRanger (v7.1.0), which resulted in 20,214 cells. During cell quality control (QC), we then removed all cells expressing $\le 1,000$ or $\ge 5,000$ genes, as well as cells with $\ge 8\%$ mitochondrial reads, with 17,653 cells remaining. We then normalized with Seurat’s *NormalizeData*, selected the top 2,000 most variable genes, and performed genewise *z*-score scaling. Next we applied PCA and performed unsupervised clustering of cells using the Louvain algorithm [27] (resolution = 0.1), based on the first 50 principal components (PCs). This resulted in 4 cell-type clusters, and the smallest cluster (with only 504 cells) was excluded from the subsequent analysis.

From the remaining 17,149 cells, we selected 18 cells for targeted amplification, 6 cells from each of the 3 remaining clusters. To select a diverse set of cells from each cluster, we used the following:

1. We identified the 2 nearest neighbors for each cell (in PCA space).
2. We excluded cells for which at least 1 nearest neighbor belonged to a different cluster.
3. For the remaining 16,295 cells, we computed the #UMI rank, from the number of observed UMIs per cell (ties were broken randomly).
4. Similarly, we computed the $\frac{\text{#UMI}}{\text{#Genes}}$ rank based on the ratio of the number of observed UMIs and the number of observed genes in the cell (ties were broken randomly).
5. Subsequently, we calculated the diversity of each cell and its neighbors as the area of the induced triangle of the cell and its neighbors in a #UMI rank × $\frac{\text{#UMI}}{\text{#Genes}}$ rank plot. The 6 cells from the 2 most diverse neighborhoods (i.e., largest triangle area) were selected for amplification.

These steps were designed to cover a diverse set of cells for which the various experimental steps had varying efficiencies. The selection of triplets from the same neighborhoods provided groups of cells with similar gene expression patterns, while the number of UMIs and the number of observed genes were used as proxies for the mRNA capture efficiencies and the health of the isolated cells.

#### Data analysis, cta-seq *ultra-deep*

The sequencing data from the *ultra-deep* sequencing run were processed using CellRanger (v7.1.0).

However, due to the high number of PCR cycles during amplification and the resulting high number of reads for the 18 selected cells, CellRanger’s UMI correction approach was no longer sufficient. Manual inspection of the reads showed that errors in the UMI sequences had inflated the number of unique reads.

This was further exacerbated by a faulty implementation of the UMI correction approach in the CellRanger software by 10X Genomics. CellRanger erroneously corrects UMIs containing sequencing errors toward other UMIs that also contain sequencing errors. For example, if we have 3 UMIs—AAAA with 10 reads, AAAT with 2 reads, and AATT with 1 read—AATT would be corrected toward AAAT (Hamming Distance 1) and stay as AAAT, even though the original 2 AAAT reads would be corrected to AAAA in the same step. We reported this issue to 10X Genomics on 13 July 2023, and 10X Genomics acknowledged the issue on 14 July 2023. The issue remains unresolved in CellRanger 7.2.0 (released on 10 November 2023).

To circumvent these issues, we extracted the relevant information for each read (count, ensemble gene ID, cell barcode, uncorrected UMI, and CellRanger-corrected UMI) from the possorted_genome_bam.bam, as provided by CellRanger, and replicated CellRanger’s read-counting workflow in R. As a sanity check, we first used the CellRanger-corrected UMIs and achieved the exact same count matrix as CellRanger. We then used the raw UMIs instead of the CellRanger-corrected UMIs, implemented the UMI tools’ directional UMI correction approach [28] in R, and applied it to correct the UMIs for the 18 selected cells, and we then counted again. The resulting count matrix showed differences for 28% of the nonzero entries when compared to the CellRanger results. We used these improved counts for the *ultra-deep* profiles in all further analysis.

### Comparison of GT and ML using cta-seq

To evaluate the performance of GT and ML based on the cta-seq dataset, we estimated the relative gene expression for the 18 selected cells by applying both estimators to the *typical* transcriptomic profiles.

The relative gene expression for the ground-truth *ultra-deep* profiles was estimated with ML. We chose ML to be conservative regarding the performance of GT and since the overestimation due to unobserved genes should be small for the *ultra-deep* profiles Supplementary Fig. S10.

#### Relative gene expression estimation

We calculated the absolute estimation error for the relative gene expression of the 18 cells by comparing the estimation results of GT and ML based on the *typical* transcriptomic profiles to the ground-truth relative gene expression of the *ultra-deep* profiles. We consider the relative gene expression estimation error of a cell to be the sum of the individual relative gene expression estimation errors in the cell.

#### Cell–cell distances

The pairwise Euclidean distances between the 18 cells were calculated in PCA space (as is common for cell–cell distances in scRNA-seq). However, to keep the necessary projections similar to a regular scRNA-seq analysis, this space could not simply be constructed based only on the 18 selected cells.

Instead, we calculated the projections based on 17,653 cells in the *typical* sequencing run. After normalization, there were 3 preprocessing steps that all depended on the context of a full dataset: variable gene selection, genewise *z*-score scaling, and PCA.

To keep these steps identical for both the GT and ML profiles of the *typical* sequenced cells, as well as the *ultra-deep* profiles, we performed them using customized functions. We used the same list of variable genes (calculated based on all 17,653 cells) for the analysis of all profiles. We then scaled the genes in all profiles using the mean and standard deviation of genes calculated based on the full 17,653 cells. Finally, we projected all profiles into the same 50 dimensional PCA space calculated from the full 17,653 cells.

In this PCA space, we calculated the pairwise distances between the ML profiles, between the GT profiles, and between the ground-truth *ultra-deep* profiles. We then compared the resulting nonzero distances based on GT and ML to the ground-truth *ultra-deep* distances.

### Comparison of GT and ML at different *UMIs/cell*

When analyzing the impact of *UMIs/cell* on the estimation performance, we used the cell with the highest number of UMIs after amplification (cell 12, cell barcode TCTCTGGGTGTGCTTA) and the cell with the second highest number of UMIs after amplification (cell 15, cell barcode GGCTTTCGTGTGTCGC).

We generated 1,000 randomly sampled profiles at each *UMI/cell* level by drawing genes from the *ultra-deep* count vector, weighted by count and with replacement. The 20 *UMI/cell* levels at which we sampled were chosen equidistant in log_10_-space from 100 to 100,000 (i.e., 100, 143, 206, 297, 428, 615, 885, 1,274, 1,832, 2,636, 3,792, 5,455, 7,847, 11,288, 16,237, 23,357, 33,598, 48,329, 69,519, 100,000 *UMIs/cell*). We then applied GT and ML, respectively, to these sampled profiles to estimate their relative gene expression.

#### Relative gene expression estimation

To assess the relative gene expression estimation performance of GT and ML, we compared their estimates for each sampled profile from cell 12 to the relative gene expression of the full *ultra-deep* profile of cell 12 and calculated the absolute error.

#### Cell–cell distance estimation

To assess cell–cell distance estimation performance, we calculated the Euclidean distances between the relative gene expression profiles of pairs of sampled profiles (either from cell 12 twice or from cell 12 and cell 15) based on GT and ML. We calculated the true distance based on the full *ultra-deep* profiles.

### Downstream analysis

#### Data analysis, pbmc3k

The pbmc3k dataset was downloaded from 10X Genomics [19] and processed following Seurat’s “Guided Clustering Tutorial” [29]. In short:

During QC, we filtered out genes expressed in fewer than 3 cells and cells with fewer than 200 expressed genes. We then filtered out cells with $>5\%$ mitochondrial reads, and finally, we removed all cells expressing more than 2,500 genes.

During preprocessing, cells were normalized using Seurat’s *NormalizeData* or *GTestimate* at default settings. For both normalization methods individually, we then identified variable genes and *z*-score scaled the data, followed by calculation of the top 10 PCs. Based on these PCs, we then constructed the neighborhood graphs and performed unsupervised Louvain clustering (resolution = 0.5). Finally, we calculated the UMAP for both conditions and annotated clusters based on marker gene expression, following the Seurat tutorial.

#### Data analysis, developing pancreas

The pancreas endocrinogenesis day 15 dataset was downloaded [30] and imported into R to be processed using Seurat. We only used the spliced counts and normalized them using *GTestimate* and *NormalizeData*; from there on, all following steps were performed identically for the 2 approaches.

First we identified variable genes and performed genewise *z*-score scaling, followed by calculation of the top 50 PCs. Based on the PCs, we constructed the neighborhood graph and performed unsupervised Louvain clustering (resolution = 0.4). Finally, we calculated the UMAP.

We manually adjusted the cluster numbering (and thereby their color) for Fig. 2C, D to have consistent cluster colors from left to right.

#### Data analysis, Spatial Transcriptomics

The stxBrain dataset of sagittal mouse brain slices from 10X Genomics was downloaded using the SeuratData R-package. In our analysis, we focused on the anterior1 slice of the dataset following Seurat’s “Analysis of spatial datasets (Sequencing-based)” vignette [31].

Our analysis differs from the vignette only in the normalization methods used. While the vignette uses *sctransform* [32] for spotwise normalization, we instead used *NormalizeData* and *GTestimate*. Direct comparison of GT and ML to *SCTransform* on the basis of relative gene expression is not possible, since *SCTransform* does not calculate relative gene expression levels. Normalization was followed by variable gene selection and genewise scaling. We then calculated the first 30 PCs and used them to construct the neighborhood graph, perform unsupervised Louvain clustering, and calculate the UMAP.

#### Data analysis, experimentally annotated PBMCs (Liu dataset)

The Liu dataset was downloaded and imported into R to be processed using Seurat. We used the purified version of the dataset, which includes an additional filtering step to ensure correct cell-type assignments.

For our *GTestimate* and *NormalizeData* analyses, we first normalized the data using the respective method at default settings, and then we identified the 2,000 most variable genes and performed genewise *z*-score scaling. For our *SCTransform*-based analysis, we simply applied *SCTransform* at its default settings, as it is supposed to replace all 3 of these steps.

From here, the remaining steps were identical for the 3 analyses: we first calculated the top 30 PCs (we chose 30 PCs to be in line with the original analysis by Fu et al. [23] performed as part of their cell filtering step) and then constructed the neighborhood graph and performed unsupervised Louvain clustering. Louvain clustering was repeated at 15 different resolutions from 0.1 to 1.5 in steps of 0.1.

At each resolution, we calculated the ARI between the experimentally annotated ground-truth cell types and the unsupervised clustering results.

## Availability of Source Code and Requirements

Project name: GTestimate

Project homepage: https://github.com/Martin-Fahrenberger/GTestimate

Operating system(s): Platform independent

Programming language: R, C++

Other Requirements: devtools, sparseMatrixStats

License: GPL3

RRID: SCR_026562

A version of record snapshot of the GitHub repository has been archived in the Software Heritage [33].

### All Code for the Analysis (raw-data, figures)

Project name: GTestimate-Paper

Project homepage: https://www.github.com/Martin-Fahrenberger/GTestimate-Paper

Operating system(s): Platform independent

Programming language: R

Other Requirements: renv (additional requirements as in notebooks/renv.lock)

License: GPL3

A version of record snapshot of the GitHub repository has been archived in the Software Heritage [34].

## Additional Files

**Supplementary Fig. S1**. Histogram showing the number of observed genes per cell for the 17,653 cells in the cta-seq sample before amplification (*typical*).

**Supplementary Fig. S2**. Raw read counts per cell before (*typical*) and after (*ultra-deep*) amplification for the 18 selected cells in the cta-seq experiment.

**Supplementary Fig. S3**. Number of observed genes before (*typical*) and after (*ultra-deep*) amplification for the 18 selected cells in the cta-seq experiment.

**Supplementary Fig. S4**. Log-normalized expression of all cell-type markers described in Seurat’s pbmc3k tutorial (zeroes not shown).

**Supplementary Fig. S5**. UMAPs visualizing the clustering of Spatial Transcriptomics spots, based on *NormalizeData* (left) and *GTestimate* (right) for the mouse brain Spatial Transcriptomics dataset.

**Supplementary Fig. S6**. Visualization of the different clusters based on *NormalizeData* (left) and *GTestimate* (right) for the mouse brain Spatial Transcriptomics dataset.

**Supplementary Fig. S7**. Similarity of the clusters based on *NormalizeData* and *GTestimate* as represented by the Jaccard Index. Clusters on the y-axis have been rearranged to maximize diagonal entries using the Hungarian algorithm.

**Supplementary Fig. S8**. *UMIs/spot* in the Spatial Transcriptomics mouse brain dataset.

**Supplementary Fig. S9**. Adjusted Rand Index (ARI) comparing unsupervised clustering results (Louvian algorithm) to the experimentally annotated cell types in the Liu dataset. Clustering was performed after normalizing with GTestimate, NormalizeData, or SCTransform and repeated for different clustering resolutions. The maximum ARI for each normalization method is indicated and labeled.

**Supplementary Fig. S10**. Missing mass before (*typical*) and after (*ultra-deep*) amplification for the 18 selected cells in the cta-seq experiment (see Suppl. Materials 1.1).

**Supplementary Fig. S11**. Histogram showing *GTestimate*’s missing mass estimates per cell for the 17,653 cells in the cta-seq sample before amplification (*typical*).

**Supplementary Table S1**. Characteristics of the regression line of the estimated vs. ground-truth distances for the cta-seq data (Fig. 1D).

**Supplementary Table S2**. PCR primer sequences used for the 18 separate cta-seq reactions. Forward primers were designed to specifically target the selected cell-barcodes and to attach the necessary sequencing adapters. Reverse primers are non-specific and also attach the necessary sequencing adapters.

giaf084_Supplemental_Files

giaf084_Authors_Response_To_Reviewer_Comments_Revision_1

giaf084_Authors_Response_To_Reviewer_Comments_Revision_2

giaf084_GIGA-D-24-00377_Original_Submission

giaf084_GIGA-D-24-00377_Revision_1

giaf084_GIGA-D-24-00377_Revision_2

giaf084_Reviewer_1_Report_Original_Submission Dr. Gregory Schwartz -- 9/30/2024

giaf084_Reviewer_1_Report_Revision_1 Dr. Gregory Schwartz -- 3/27/2025

giaf084_Reviewer_2_Report_Original_Submission Dr. Amichai Painsky -- 12/15/2024

## Abbreviations

ARI: Adjusted Rand Index; cta-seq: cell-targeted PCR amplification followed by sequencing; GT: Good–Turing estimator; ML: maximum likelihood estimator; PC: principal component; scRNA-seq: single-cell RNA sequencing.

## Data Availability

Processed cta-seq data are available in NCBI via GEO accession number GSE268930. Raw sequencing data are available via controlled access at the European Genome-Phenome Archive (EGA) under accession number EGAD50000001338.
