# Supplementary material for: GTestimate: improving relative gene expression estimation in scRNA-seq using the Good–Turing estimator
Source: Gigascience. 2025 Oct 8;14:giaf084. doi: 10.1093/gigascience/giaf084 (PMC12569601; doi:10.1093/gigascience/giaf084)
Supplement: giaf084_Supplemental_Files [file giaf084_supplemental_files.zip › Main_v2_for Supplement.pdf]

1 Supplementary Materials

1.1 The Missing Mass

Besides improving the relative expression estimates of observed genes, GT can also estimate the sum of the relative frequencies of all unobserved genes. This can be viewed as the probability p_0 that a next hypothetical UMI would be of a currently unobserved gene. We have therefore termed p_0 the missing-mass of the relative gene expression distribution.

The missing-mass for each cell is estimated from the number of genes with a UMI count of one (N_1) and the sum of all counts ($\sum_g c_g$) as has previously been discussed [16, 17].

$$\widehat{p_0} = \frac{N_1}{\sum_g c_g} \tag{S1}$$

When applied to a Seurat or SingleCellExperiment object in R *GTestimate* saves the estimated $\widehat{p_0}$ for each cell into a meta-data vector called "missing_mass".
The Simple Good-Turing estimator scales the relative frequencies (including p_0) to ensure

$$\sum_g \widehat{f_g}^{GT} + \widehat{p_0} = 1 \tag{S2}$$

for each cell.

Equation S1 provides insight into the amount of information present for each cell, which may warrant further study. E.g. the missing-mass in the cta-seq experiment is substantially reduced after cell targeted amplification of reads (Fig. S10).

Due to the typically low *UMIs/cell*, this missing mass of a cell in scRNA-seq can be quite substantial (Fig. S11).

1.2 Supplementary Tables

Method	Slope	Sum of absolute Residuals	Intercept	Sum of absolute Errors
ML	1.529	1511.317	0.955	3258.049
GT	1.302	1263.276	-0.408	2093.645

Table S1: Characteristics of the regression line of the estimated vs. ground-truth distances for the cta-seq data (Fig. 1d).

1.3 Supplementary Figures

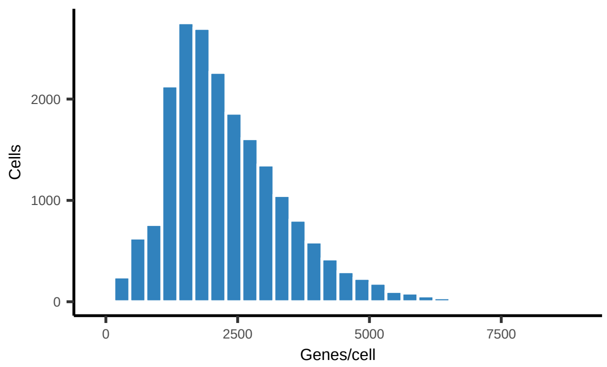

Figure S1: Histogram showing the number of observed genes per cell for the 17,653 cells in the cta-seq sample before amplification (*typical*).

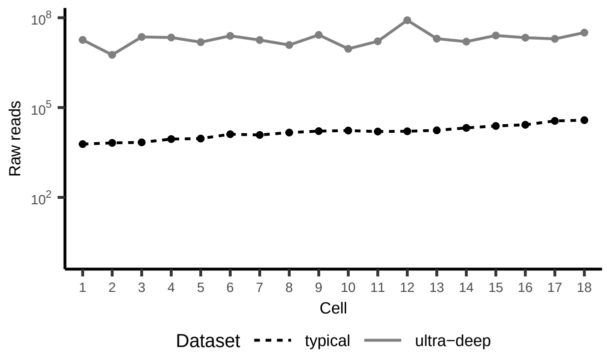

Figure S2: Raw read counts per cell before (*typical*) and after (*ultra-deep*) amplification for the 18 selected cells in the cta-seq experiment.

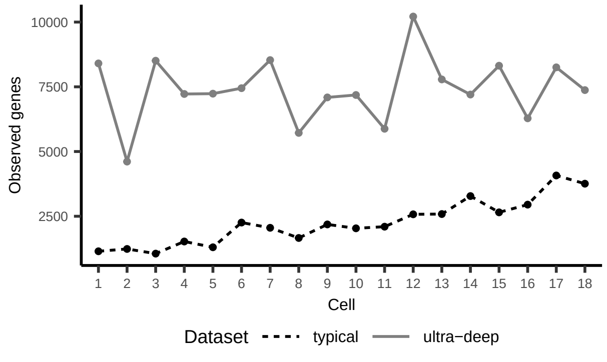

Figure S3: Number of observed genes before (*typical*) and after (*ultra-deep*) amplification for the 18 selected cells in the cta-seq experiment.

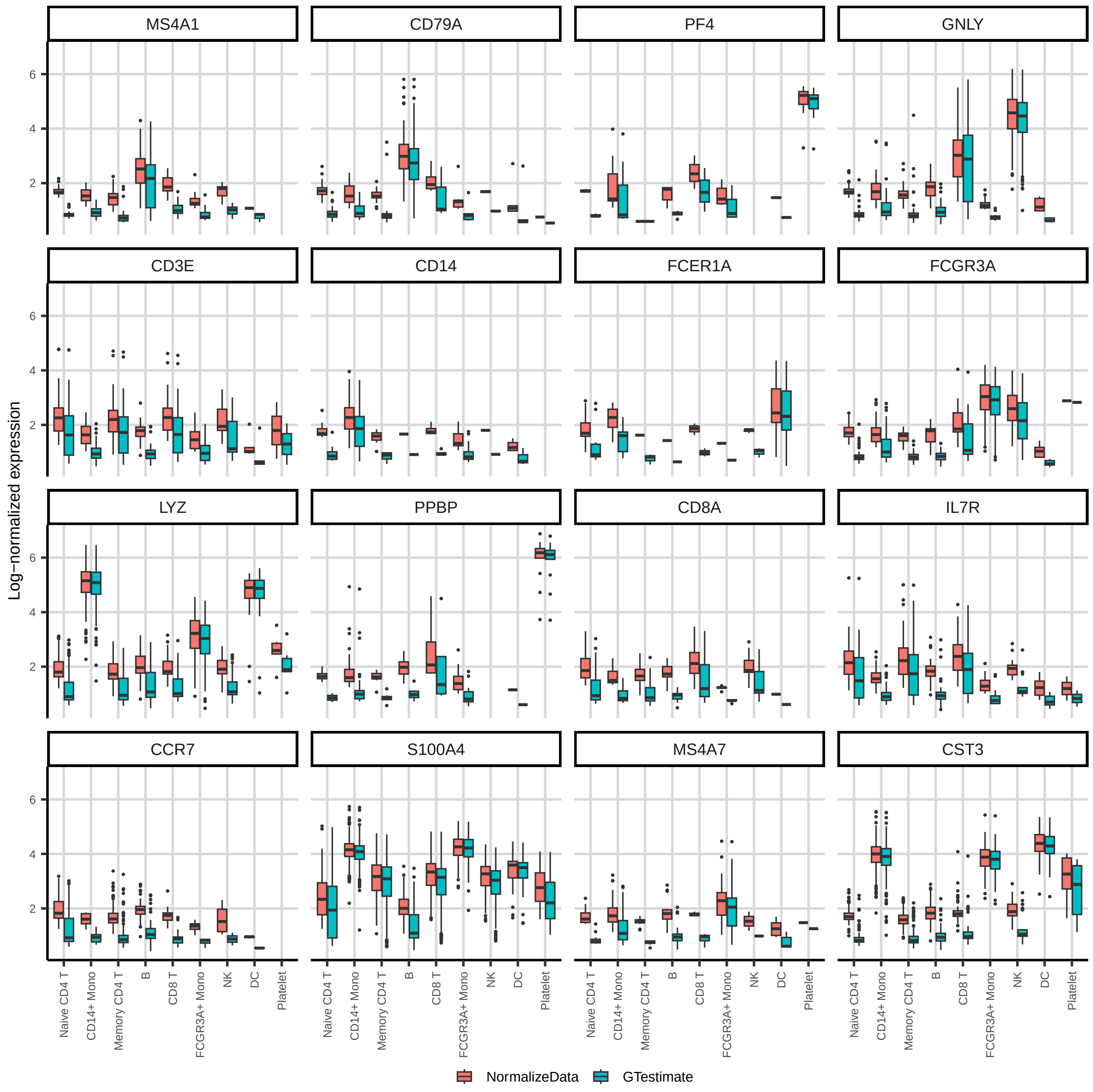

Figure S4: Log-normalized expression of all cell-type markers described in Seurat's pbmc3k tutorial (zeroes not shown).

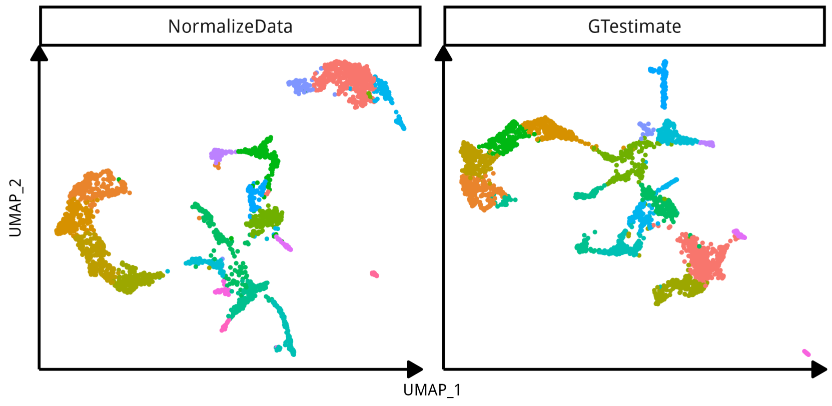

Figure S5: UMAPs visualizing the clustering of Spatial Transcriptomics spots, based on *NormalizeData* **(left)** and *GTestimate* **(right)** for the mouse brain Spatial Transcriptomics data-set.

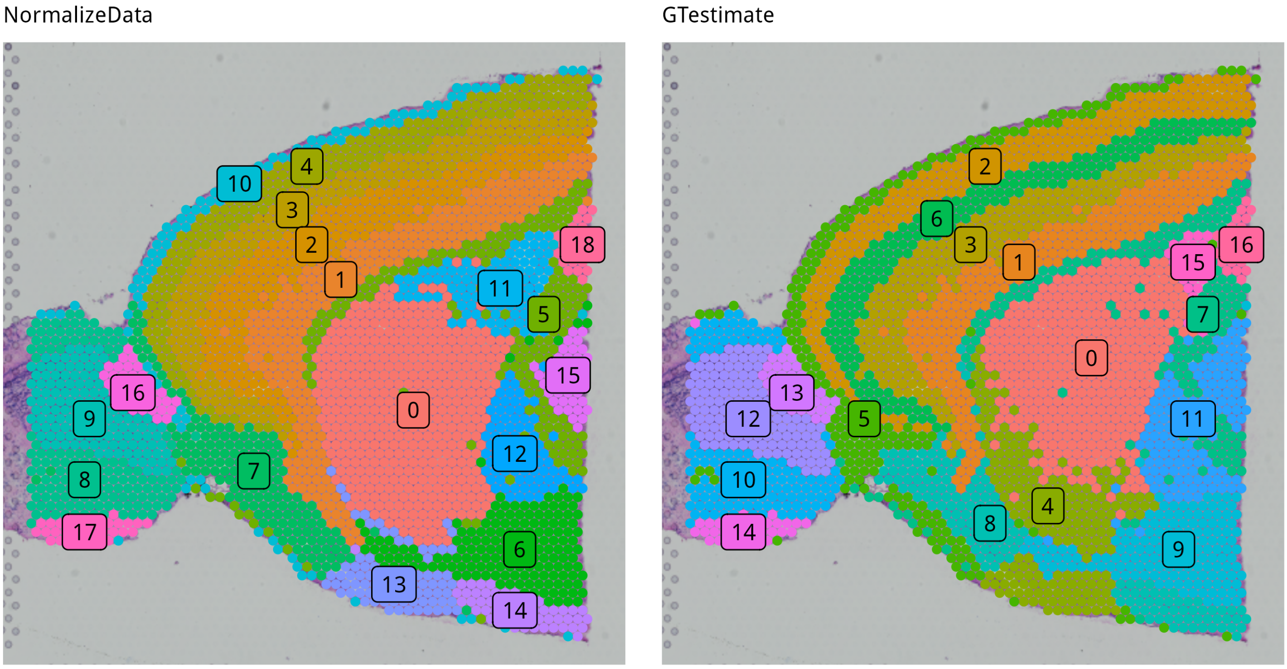

Figure S6: Visualization of the different clusters based on *NormalizeData* **(left)** and *GTestimate* **(right)** for the mouse brain Spatial Transcriptomics data-set.

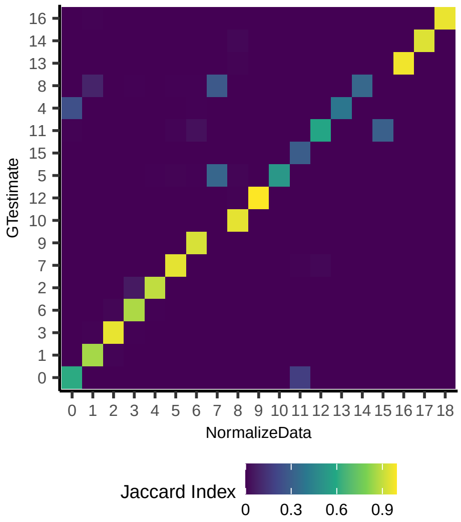

Figure S7: Similarity of the clusters based on *NormalizeData* and *GTestimate* as represented by the Jaccard Index. Clusters on the y-axis have been rearrange to maximize diagonal entries using the Hungarian Algorithm.

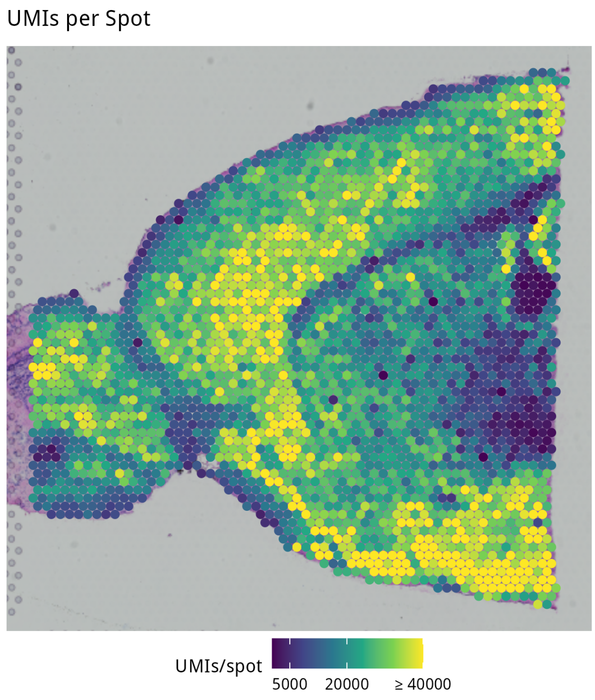

Figure S8: *UMIs/spot* in the Spatial Transcriptomics mouse brain data-set.

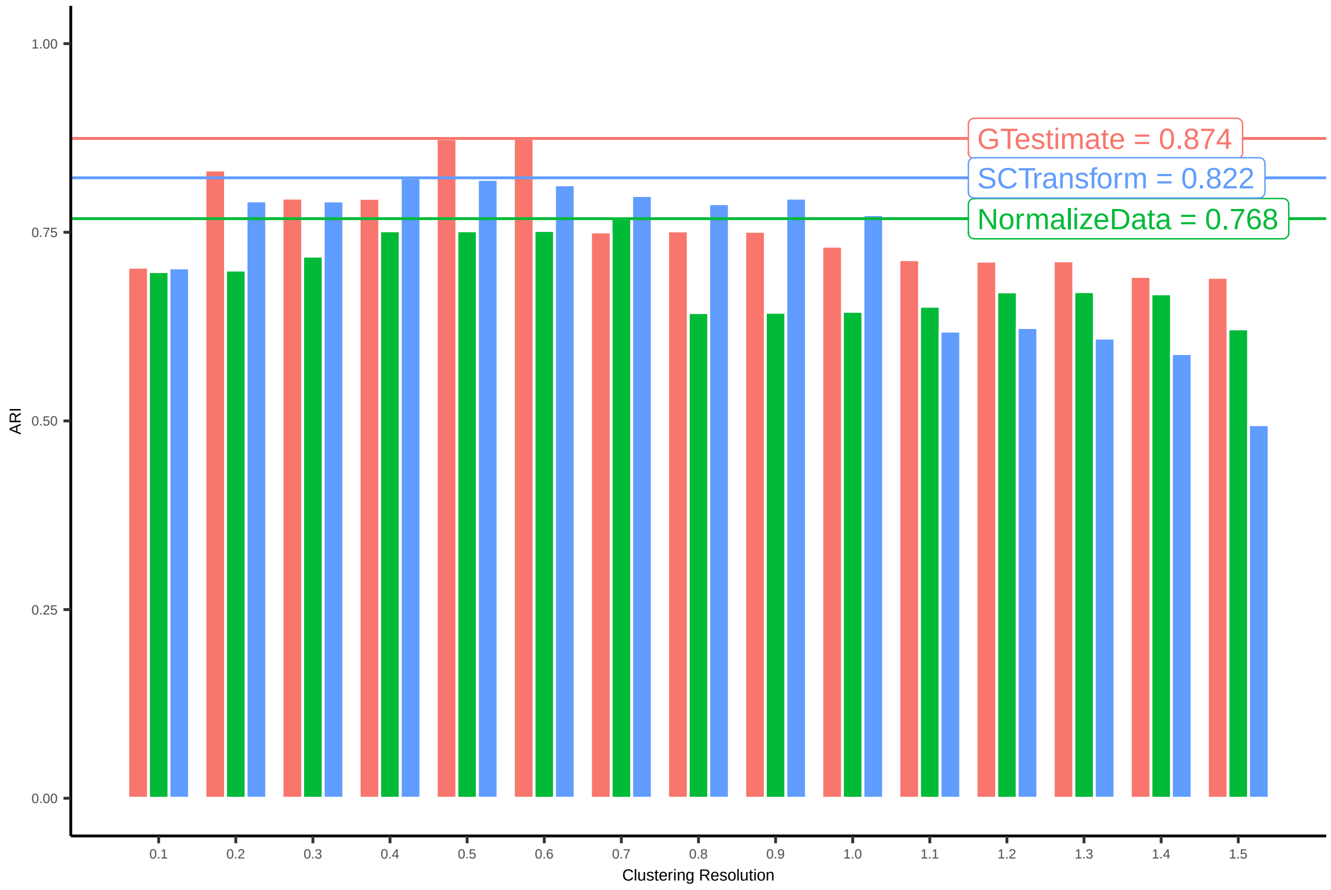

Figure S9: Adjusted Rand Index (ARI) comparing unsupervised clustering results (Louvian algorithm), to the experimentally annotated cell-types in the liu data-set. Clustering was performed after normalizing with either GTestimate, NormalizeData or SCTransform and repeated for different clustering resolutions. The maximum ARI for each normalization method is indicated and labeled.

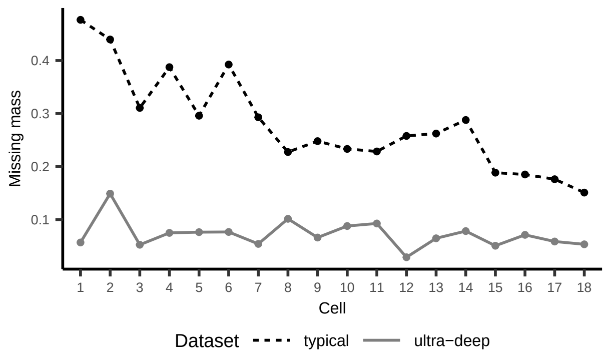

Figure S10: Missing mass before (*typical*) and after (*ultra-deep*) amplification for the 18 selected cells in the cta-seq experiment (see Suppl. Materials 1.1).

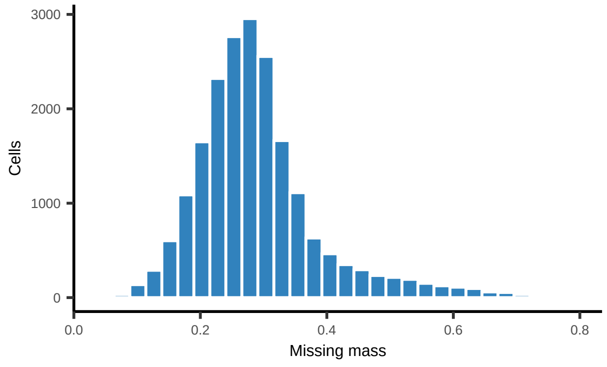

Figure S11: Histogram showing *GTestimate*'s missing mass estimates per cell for the 17,653 cells in the cta-seq sample before amplification (*typical*).
