# Supplementary material for: GTestimate: improving relative gene expression estimation in scRNA-seq using the Good–Turing estimator
Source: Gigascience. 2025 Oct 8;14:giaf084. doi: 10.1093/gigascience/giaf084 (PMC12569601; doi:10.1093/gigascience/giaf084)
Supplement: giaf084_GIGA-D-24-00377_Revision_1 [file giaf084_giga-d-24-00377_revision_1.pdf]

GigaScience
GTestimate: Improving relative gene expression estimation in scRNA-seq using the Good-Turing estimator
--Manuscript Draft--

**Manuscript Number:**	GIGA-D-24-00377R1
**Full Title:**	GTestimate: Improving relative gene expression estimation in scRNA-seq using the Good-Turing estimator
**Article Type:**	Technical Note
**Funding Information:**	H2020 Marie Skłodowska-Curie Actions (H2020-MSCA-ITN-2017-765104) — Univ.-Prof. Dr. Arndt von Haeseler<br>Österreichischen Akademie der Wissenschaften (F78) — Univ.-Prof. Dr. Arndt von Haeseler
**Abstract:**	Background: Single-cell RNA-seq suffers from unwanted technical variation between cells, caused by its complex experiments and shallow sequencing depths. Many conventional normalization methods try to remove this variation by calculating the relative gene expression per cell. However, their choice of the Maximum Likelihood estimator is not ideal for this application.<br>Results: We present GTestimate, a new normalization method based on the Good-Turing estimator, which improves upon conventional normalization methods by accounting for unobserved genes.<br>To validate GTestimate we developed a novel cell targeted PCR-amplification approach (cta-seq), which enables ultra-deep sequencing of single cells.<br>Based on this data we show that the Good-Turing estimator improves relative gene expression estimation and cell-cell distance estimation.<br>Finally, we use GTestimate's compatibility with Seurat workflows to explore four example data-sets and show how it can improve downstream results.<br>Conclusion: By choosing a more suitable estimator for the relative gene expression per cell, we were able to improve scRNA-seq normalization, with potentially large implications for downstream results. GTestimate is available as an easy-to-use R-package and compatible with a variety of workflows, which should enable widespread adoption.
**Corresponding Author:**	Martin Fahrenberger<br>Center for Integrative Bioinformatics Vienna (CIBIV)
**Corresponding Author Secondary Information:**	
**Corresponding Author's Institution:**	Center for Integrative Bioinformatics Vienna (CIBIV)
**Corresponding Author's Secondary Institution:**	
**First Author:**	Martin Fahrenberger
**First Author Secondary Information:**	
**Order of Authors:**	Martin Fahrenberger<br>Christopher Esk<br>Jürgen Arthur Knoblich<br>Arndt von Haeseler
**Order of Authors Secondary Information:**	
**Response to Reviewers:**	Summary:<br>We thank both reviewers for their valuable comments, which helped improve our manuscript. Based on their suggestions, we've made several key revisions:<br>Included additional comparisons with SCTransform through a new clustering analysis using an experimentally labeled dataset (Fig S9).<br>Clarified the Good-Turing estimator formula by explicitly including smoothing,

Powered by Editorial Manager® and ProduXion Manager® from Aries Systems Corporation

addressing methodological transparency concerns.
Provided clearer justifications for methodological choices and corrected figure referencing issues.
These revisions have substantially enhanced the clarity and robustness of our manuscript.
As per Journal guidance we have registered GTestimate at bio.tools and SciCrunch.org (l. 360).
Detailed responses to Reviewer comments can be found below.

Reviewer 1:

We thank Reviewer 1 for their thoughtful and constructive comments, which have significantly improved our manuscript. Below, we address the comments point-by-point.

In this manuscript, Fahrenberger et al. propose a new scRNA-seq normalization method to more accurately report UMI counts of individual cells. They specifically use a Good-Turing estimator, compared with a more commonly used Maximum Likelihood estimator, to adjust raw UMI counts. Using their own cta-seq, a cell targeted PCR-amplification strategy, as ground truth, they compare their estimator with a traditional size-corrected estimator. Furthermore, they illustrate downstream changes using their method, including changes to clustering results and spatial transcriptomic readouts. The manuscript was a clear read and presents an interesting alternative solution to an often overlooked, but important, problem. However, there are some aspects of the manuscript that need to be addressed.

R1C1:
Some major content missing includes comparisons with more widely-used normalization methods throughout the manuscript, and better ground truth data sets in their downstream analysis.

Author Response:
We appreciate this comment, however, we purposefully limited the comparisons to NormalizeData to keep all steps, other than the relative gene expression estimation, unchanged (Fig 1b). Our improved relative gene expression estimation is directly transferable to other methods such as scran's computeSumFactors or scanpy's normalize_total.

Direct comparisons to methods such as SCTransform, which involve additional data transformations, are only possible based on downstream results. Here, a lack of generally accepted ground-truth data-sets makes fair comparisons nearly impossible. One of the reasons why we designed cta-seq was to gain the best possible ground-truth data-set. We have now added to the main text (l. 104-109) to more clearly justify why methods such as e.g. SCTransform are not directly comparable.

We have also added clustering based analysis of a recent data-set which includes ground-truth cell-type annotations (l. 141-164 and Figure S9 and Methods l. 338-350). For this data-set we included SCTransform in our comparison. These results further highlight the strengths of GTestimate.

Specific comments are as follows:

R1C2:
l. 34: To my knowledge, most groups do not use a single division by total UMI count as the only normalization. Seurat has NormalizeData, but also heavily promotes scTransform, a completely different method. Many use log transform (as I believe was done here), some use quantile transform, others use regression techniques etc. It was odd to see these standard normalizations missing in comparisons. The authors should use such standard procedures to demonstrate the superiority of GT.

Author Response:
Many other scRNA-seq normalization methods apply additional transformations to the data or do not compute relative gene expression values. In the manuscript, we state that the calculation of relative gene expression with ML is inherent to all global-scaling methods (l. 33). Our comparison is specifically focused on improving the initial

estimation for these methods, while keeping all other steps unchanged.

Regarding the usage frequency of different normalization methods: A look at recent scRNA-seq publications shows that "NormalizeData" and "SCTransform" are used roughly equally.
Additionally, Ahlmann-Eltze and Huber (2023) performed a large comparison of scRNA-seq data transformations/normalizations. They concluded that “delta methods” such as NormalizeData are as good as or better than other approaches, further justifying our focus on NormalizeData as a comparison.

R1C3:
l. 42: Is there a justification for the successor function being applied within the frequency ((cg + 1) / total) instead of outside ((cg / total) + 1) as is expected with the Good-Turing estimation?

Author Response:
We have checked the formula again, it follows from equations 2 and 2’ in Good (1953).

R1C4:
Furthermore, there is typically a smoothing function for erratic N_cg values, which I would expect with single-cell data. In the methods there is a brief mention of linear smoothing, but that would imply that the GT equation is
misleading and oversimplified. The actual equation should be included in the main text to avoid confusion.

Author Response:
We referred to our estimator as a Simple Good Turing estimator in the main text and cited [3] to indicate the use of the smoothing procedure.
To clarify, we now explicitly included the smoothing function S() in our main equation (GT) and added an additional reference to the smoothing procedure described by Gale and Sampson (1995) chapter 4 (l. 42-44). We believe this addition resolves potential confusion and enhances methodological transparency.

R1C5:
l. 58: Compared to 16,965 reads average per cell, what is the equivalent for the ultra-deep sequencing (not 23 million reads, as that is not 7.4 fold increase)?

Author Response:
The 7.4 fold increase is the ratio of, on average, 44,511 UMIs/cell (ultra-deep) to, on average, 6,048 UMIs/cell (typical). The 23 million reads/cell and the 16,965 reads/cell are the corresponding average numbers of raw reads per condition before UMI based deduplication.
This is now more clearly expressed (l. 59).

R1C6:
I am not entirely convinced on the use of cta-seq as a ground-truth for the cells, especially in comparison with ML. The authors should show that cta-seq has similar UMI and gene count distributions to more popular scRNA-seq technologies (e.g. 10x Chromium) or the application may be specific to cta-seq only.

Author Response:
We are uncertain which aspect of cta-seq Reviewer 1 is unconvinced by.

To clarify:
Our “typical” sample is a standard 10X Chromium data-set. We essentially used leftover material from the same 10X Chromium library prep for our “ultra-deep” sample. Fig 1a and Fig S1 show the UMI and gene count distribution of the “typical” sample, which is similar to other 10X Chromium data-sets.

The “ultra-deep” sample was generated through targeted amplification of the leftover material, using primers specific to the 18 selected cell-barcodes. The targeted PCR amplifications were performed in 18 separate reactions. Therefore, PCR-efficiency biases, due to the different primers, would affect all cDNAs from the same cell equally. (We have adjusted the methods section to more clearly state the 18 separate PCR

Powered by Editorial Manager® and ProduXion Manager® from Aries Systems Corporation

reactions. (l. 204-205))

This leaves potential differences in efficiency between the cells in our “ultra-deep” sample, these are present as we can see in Fig S2. However, they are mitigated through the UMI based deduplication of reads and by the fact that we only use relative gene expression levels in all our comparisons.

Assessment of the “ultra-deep” data based on gene-count or UMI count distributions is not possible due to the low number of only 18 cells.

R1C7:
l. 110: Instead of using unknown classification data sets, there are existing cell-sorted data sets with ground truths (many even on the 10x website). The authors should use these data sets to compare downstream analysis.

Author Response:
After some additional literature research we identified the liu data-set from Fu et al. 2024 as a good candidate for an experimentally labeled scRNA-seq data-set.

We used this data-set to compare GTestimate, NormalizeData and also SCTransform based on the Adjusted Rand Index (ARI) of the clustering results.
Our results show that GTestimate achieves the highest overall ARI at 0.874 compared to SCTransform’s 0.822 and NormalizeData’s 0.768.

We have added Figure S9 and lines 141-164 in the results section, as well as lines 338-350 in the Methods section.

R1C8:
l. 125: The spatial transcriptomic results were very subjective, with no statistical hypotheses.

Author Response:
The results in Fig 2e are mostly a proof-of-principle, highlighting an additional application for GTestimate.
Additionally, they highlight the differences in downstream results when using GTestimate instead of NormalizeData.

R1C9:
The entire manuscript is missing any sort of statistics when comparing methods, which is a major flaw and should be rectified.

Author Response:
As explained in R1C1 the lack of ground-truth prohibits the use of statistics to compare downstream results.
We specifically designed cta-seq to gain ground-truth relative gene expression values and for this data the results are quite clear. Fig 1d, for example, shows that GT outperforms ML in 18 of 18 cells.

At the reviewer’s request we added Fig S9, which now provides an additional comparison based on experimentally annotated cell-types.

Our results clearly indicate the superiority of GTestimate.

R1C10:
Here specifically, the color scale stops at 3, but does this carry over to the relative differential expression?

Author Response:
To compute the relative difference in gene expression we used the full range of log-normalized gene expression values.
For illustration purposes we only show the log-normalized gene expression difference between 0 and 3 to highlight the difference for the lowly expressed spots, which are

Powered by Editorial Manager® and ProduXion Manager® from Aries Systems Corporation

mostly affected by the switch from ML to GT.

R1C11:
The claim is that it is constant, but if they are all greater than 3 then they must be quite variable, so it is surprising to see such a constant value of 0. Maybe the complete color scale should be shown on all figures to clarify this.

Author Response:
The choroid plexus (red area) is characterized by high expression values, in such situations the relative difference between NormalizedData and GTestimate is small (as illustrated in Fig 2f), thus, it is not surprising that the differences all appear to be close to zero.

R1C12:
From my understanding of the manuscript, the 18 cells for analysis and comparison were chosen based on a typical Seurat analysis. This technique introduces a range of biases into the comparison and makes the argument a bit circular.

For a bias example, the top 2000 most variable genes were used, suggesting that entire classes of genes may be ignored even when highly or lowly expressed, such as housekeeping genes.

There also appears to be many steps that were not entire justified outside of a "typical analysis", for example excluding a cluster in the analysis (just because it was not that large?), only selection 18 cells (why 6 from each cluster?), removing cells with less than 1000 expressed genes or over 8% mitochrondrial reads (this may be an issue, and removing specific cell types or proliferating cells, this should be a bivariate removal with justification). All of these filterings remove generalizeability of GT.

Author Response:
We appreciate the reviewer raising this important concern. To clarify, the choice of cells used for benchmarking GT versus ML was intended purely to ensure diversity among the selected cells, rather than to achieve optimal results for any particular normalization method. In fact, the exact selection of these 18 cells (e.g., choosing 6 cells per cluster or excluding small clusters) is relatively unimportant; selecting 18 cells at random would also have been a valid approach. The key purpose was to include a diverse collection of cell types with differing transcriptional profiles, spanning both low quality and high quality cells. This ensures robustness of our conclusions across both biological and technical variance.

We chose 18 cells as this seemed to be a large enough set to draw conclusions without diluting our sequencing efforts among too many cells.

Regarding the filtering steps applied (e.g., exclusion of cells with fewer than 1000 expressed genes or those exceeding 8% mitochondrial reads), we followed common quality-control practices frequently used in standard scRNA-seq analyses (https://satijalab.org/seurat/articles/pbmc3k_tutorial). Our intention was to adopt a transparent, standard workflow that readers can easily replicate.

The generalizability of GTestimate is not impacted by the data-set chosen, since GTestimate was not used during cell selection for cta-seq, and cell selection was not optimized to favor GTestimate.

R1C13:
Supplementary Figures in the text hyperlink to the main figures which is confusing. More importantly, the caption of Supplementary Figures read "Figure" rather than "Supplementary Figures".

Author Response:
This has been corrected in the revised manuscript.

Powered by Editorial Manager® and ProduXion Manager® from Aries Systems Corporation

Reviewer 2:

We thank Reviewer 2 for the comments. We hope our point-by-point response alleviates Reviewer 2’s concerns.

This paper introduces a Good-Turing (GT) estimation scheme for relative gene expression estimation and cell-cell distance estimation. The proposed methods, namely GTestimate, claims to improve upon conventional normalization methods by accounting for unobserved genes.
The idea behind this contribution is fairly straightforward - since the relative gene expression is of large alphabet, a GT estimator is expected to preform better than a naive ML approach. However, I am not convinced that the authors applied it correctly.

R2C1:
First, the proposed GT estimator (as appears in (GT)) in the text), assigns a zero estimate to unobserved genes (Cg = 0). This contradicts the entire essence of using a GT estimator.

Author Response:
Our decision to assign zero estimates to unobserved genes (where C_g = 0) was deliberate and justified by biological considerations and does not contradict the essence of Good-Turing estimation.
Count-matrices in scRNA-seq typically contain 20,000-30,000 genes to accommodate all expressed genes across the different cell-types. However, current understanding of biology tells us that any individual cell type expresses only a subset of these genes. This creates a scenario where:
The true alphabet size (number of genuinely expressed genes per cell type) is unknown and varies between cell types
We perform thousands of individual GT estimations (one per cell)
There is no reliable way to determine which specific unobserved genes should receive portions of the missing mass in the current cell
Gale and Sampson (1995) [3] acknowledged that GT estimators can not by themselves redistribute the missing mass in §9 of their paper: "Good-Turing techniques give an overall estimate P_0 for the probability of all unseen species taken together, but in themselves they can give no guide to the individual probabilities of the separate unseen species.”
They further note: “Provided the number of unseen species is known, the obvious approach is to divide P_0 equally between the species. But this is a very unsatisfactory technique."
They then go on to discuss a data-set specific approach to the problem, which can not be transferred to scRNA-seq data-sets. Developing a suitable way to redistribute the missing mass in scRNA-seq data-sets remains an open problem which may warrant further research.

We also did not want to assign small non-zero expression values to all unobserved genes, as this may introduce unpredictable effects across the various downstream analysis tools and violate their assumptions.

R2C2:
Second, it makes no since to use this expression for every Cg > 0. In fact, any reasonable GT based estimator applies GT for relatively small Cg, and ML estimator for large Cg. See [1] for a through discussion. The choice of a threshold between "small" and "large" Cg's is subject to many studied (for example [2], [1]), but it makes no sense to use the above expression for any Cg.

Author Response:
This comment is a bit surprising to us, since many of the seminal papers on GT estimators suggested using the same GT expression independent of C_g.

After this remark we reexamined the methods introduced in [1] and [2].
The two papers introduce a combined ML and GT estimator with a switching-rule to decide which estimate is used. Drukh et al. suggest using the GT estimate for any C_g

<= N^(2/5) and the ML estimate for C_g > N^(2/5), where N is the sample size. However, we remain unconvinced of the usefulness of such an estimator for our data-sets for two reasons:

First, there are only very few genes per cell for which this switching rule comes into effect. When tested on the pbmc3k data-set (also used in the paper), a quick analysis revealed that, on average, the switching rule would use ML instead of GT for 13.34 genes per cell out of the, on average, 849 expressed genes.

Second, by definition this rule would only apply for the highest C_g values while, at the same time, the relative difference between the ML estimates and GT estimates gets smaller for higher values C_g. This indicates that a switch between the two methods will only have marginal effects. For the pbmc3k data-set the affected genes, on average, showed an absolute relative difference between the GT and the ML estimate of 0.024, confirming that a combined estimator would only make small adjustments.

R2C3:
Finally, notice that if N_{Cg} > 0 for some g but N_{Cg+1} = 0, the proposed estimator is not defined. There exists several smoothing solutions for such cases (for example [3]), but they need to be properly discussed.

Author Response:
We referred to our estimator as a Simple Good Turing estimator in the main text and cited [3]. We also discussed the need for smoothing of the N_{C_g} values in the methods section (l. 183-187).
To further clarify, we now explicitly included the smoothing function S() within our main equation (GT) and added an additional reference to the smoothing procedure described by [3] (l. 42-44). We believe this addition resolves potential confusion and enhances methodological transparency.

R2C4:
to conclude, I am not sure what is the effect of these issues on the experiments in the paper, which makes it difficult to assess the results.

Author Response:
We hope that our point-by-point responses above lay any concerns to rest and that the results in our paper now speak for themselves.

REFERENCES

[1] A. Painsky, "Convergence guarantees for the good-turing estimator," Journal of Machine Learning Research,
vol. 23, no. 279, pp. 1-37, 2022.
[2] E. Drukh and Y. Mansour, "Concentration bounds for unigram language models." Journal of Machine Learning
Research, vol. 6, no. 8, 2005.
[3] W. A. Gale and G. Sampson, "Good-Turing frequency estimation without tears," Journal of quantitative
linguistics, vol. 2, no. 3, pp. 217-237, 1995.

Additional Information:	
**Question**	**Response**
Are you submitting this manuscript to a special series or article collection?	No
**Experimental design and statistics**<br><br>Full details of the experimental design and statistical methods used should be given in the Methods section, as detailed in our	Yes

Powered by Editorial Manager® and ProduXion Manager® from Aries Systems Corporation

Minimum Standards Reporting Checklist. Information essential to interpreting the data presented should be made available in the figure legends.<br><br>Have you included all the information requested in your manuscript?	
**Resources**<br><br>A description of all resources used, including antibodies, cell lines, animals and software tools, with enough information to allow them to be uniquely identified, should be included in the Methods section. Authors are strongly encouraged to cite Research Resource Identifiers (RRIDs) for antibodies, model organisms and tools, where possible.<br><br>Have you included the information requested as detailed in our Minimum Standards Reporting Checklist?	Yes
**Availability of data and materials**<br><br>All datasets and code on which the conclusions of the paper rely must be either included in your submission or deposited in publicly available repositories (where available and ethically appropriate), referencing such data using a unique identifier in the references and in the "Availability of Data and Materials" section of your manuscript.<br><br>Have you have met the above requirement as detailed in our Minimum Standards Reporting Checklist?	No
If not, please give reasons for any omissions below.<br><br>as follow-up to "**Availability of data and materials**	Raw data for the cta-seq experiment is not yet available due to patient privacy concerns. It will be made available through controlled access at EGA shortly.

All datasets and code on which the conclusions of the paper rely must be either included in your submission or deposited in publicly available repositories (where available and ethically appropriate), referencing such data using a unique identifier in the references and in the “Availability of Data and Materials” section of your manuscript.<br><br>Have you have met the above requirement as detailed in our Minimum Standards Reporting Checklist?<br><br>"	

Main Manuscript Click here to access/download;Manuscript;GTestimate.pdf

GTestimate: Improving relative gene expression estimation in scRNA-seq using the Good-Turing estimator

Martin Fahrenberger *[1,2], Christopher Esk[3,4], Jürgen A. Knoblich[4,5], and Arndt von Haeseler[6]

[1]Center for Integrative Bioinformatics Vienna (CIBIV), Max Perutz Labs, University of Vienna and Medical University of Vienna, Vienna BioCenter (VBC), Vienna, Austria.
[2]Vienna Biocenter PhD Program, a Doctoral School of the University of Vienna and the Medical University of Vienna, Vienna, Austria.
[3]Institute of Molecular Biology, University of Innsbruck, Innsbruck, Austria
[4]Institute of Molecular Biotechnology of the Austrian Academy of Science (IMBA), Vienna BioCenter (VBC), Vienna, Austria
[5]Department of Neurology, Medical University of Vienna, Vienna, Austria.
[6]Ludwig Boltzmann Institute for Network Medicine, University of Vienna, Vienna, Austria

March 25, 2025

Abstract

Background: Single-cell RNA-seq suffers from unwanted technical variation between cells, caused by its complex experiments and shallow sequencing depths. Many conventional normalization methods try to remove this variation by calculating the relative gene expression per cell. However, their choice of the Maximum Likelihood estimator is not ideal for this application.

Results: We present *GTestimate*, a new normalization method based on the Good-Turing estimator, which improves upon conventional normalization methods by accounting for unobserved genes. To validate *GTestimate* we developed a novel cell targeted PCR-amplification approach (cta-seq), which enables ultra-deep sequencing of single cells. Based on this data we show that the Good-Turing estimator improves relative gene expression estimation and cell-cell distance estimation. Finally, we use *GTestimate*'s compatibility with Seurat workflows to explore four example data-sets and show how it can improve downstream results.

Conclusion: By choosing a more suitable estimator for the relative gene expression per cell, we were able to improve scRNA-seq normalization, with potentially large implications for downstream results. *GTestimate* is available as an easy-to-use R-package and compatible with a variety of workflows, which should enable widespread adoption.

*martin.fahrenberger@gmail.com

Keywords

scRNA-seq, Normalization, Gene Expression, Good-Turing estimator, Deep Sequencing, Targeted Amplification

Introduction

Single-cell RNA-seq (scRNA-seq) provides new insights into cell diversity, differentiation and disease [1, 2, 3]. These insights are enabled by affordable high-throughput methods for the parallel sequencing of thousands of cells [4, 5]. However, they require many experimental steps, whose efficiency differs between cells, leading to high variability in the number of mRNAs captured. Additionally, sequencing depths as low as 20,000 reads per cell [6] and the nature of parallel sequencing introduce stochastic variation [5, 7, 8]. After accounting for PCR-duplicates among reads, a median of ∼5,000 *UMIs/cell* (number of sequenced mRNA molecules per cell) with a range of ∼500-20,000 *UMIs/cell* is typical for a high quality sample (Figure 1a). This high technical variation between cells results in a low signal-to-noise ratio, which makes data analysis challenging.

During data processing (Figure 1b) *global-scaling normalization* methods [8] such as e.g. Seurat's *NormalizeData* [9], scran's *computeSumFactors* [10, 11] or scanpy's *normalize_total* [12] account for the variation in *UMIs/cell* by calculating a single scaling-factor (or size-factor) per cell. Despite its simplicity, this approach has been shown to outperform more complex methods [13].

Global-scaling normalization inherently requires the calculation of the relative gene expression levels per cell. Although not typically discussed as such, the calculation used by these methods is a Maximum Likelihood estimation (ML) [14] of the relative gene expression frequency per cell.

$$\widehat{f_g}^{ML} = \frac{c_g}{\sum_i c_i} \tag{ML}$$

where c denotes the transcriptomic profile of the cell with a count c_g for each gene g.

However, at ∼5,000 *UMIs/cell* only ∼2.5% of the ∼200,000 mRNA transcripts in a typical mammalian cell [15] are sequenced and many expressed genes remain unobserved, as evident by the low *genes/cell* observed in scRNA-seq experiments (Figure S1). ML then estimates the relative expression of unobserved genes as zero. This inherently leads to overestimation of the relative expression for observed genes, since the sum of all relative frequencies equals one ($\sum_g \widehat{f_g}^{ML} = 1$).

To reduce this overestimation we propose a Simple Good-Turing estimator [16, 17]. R1C4

R2C3

$$\widehat{f_g}^{GT} = \begin{cases} \frac{(c_g+1)}{\sum_i c_i} \cdot \frac{S(N_{c_g+1})}{S(N_{c_g})}, & \text{for } c_g > 0 \\ 0, & \text{for } c_g = 0 \end{cases} \tag{GT}$$

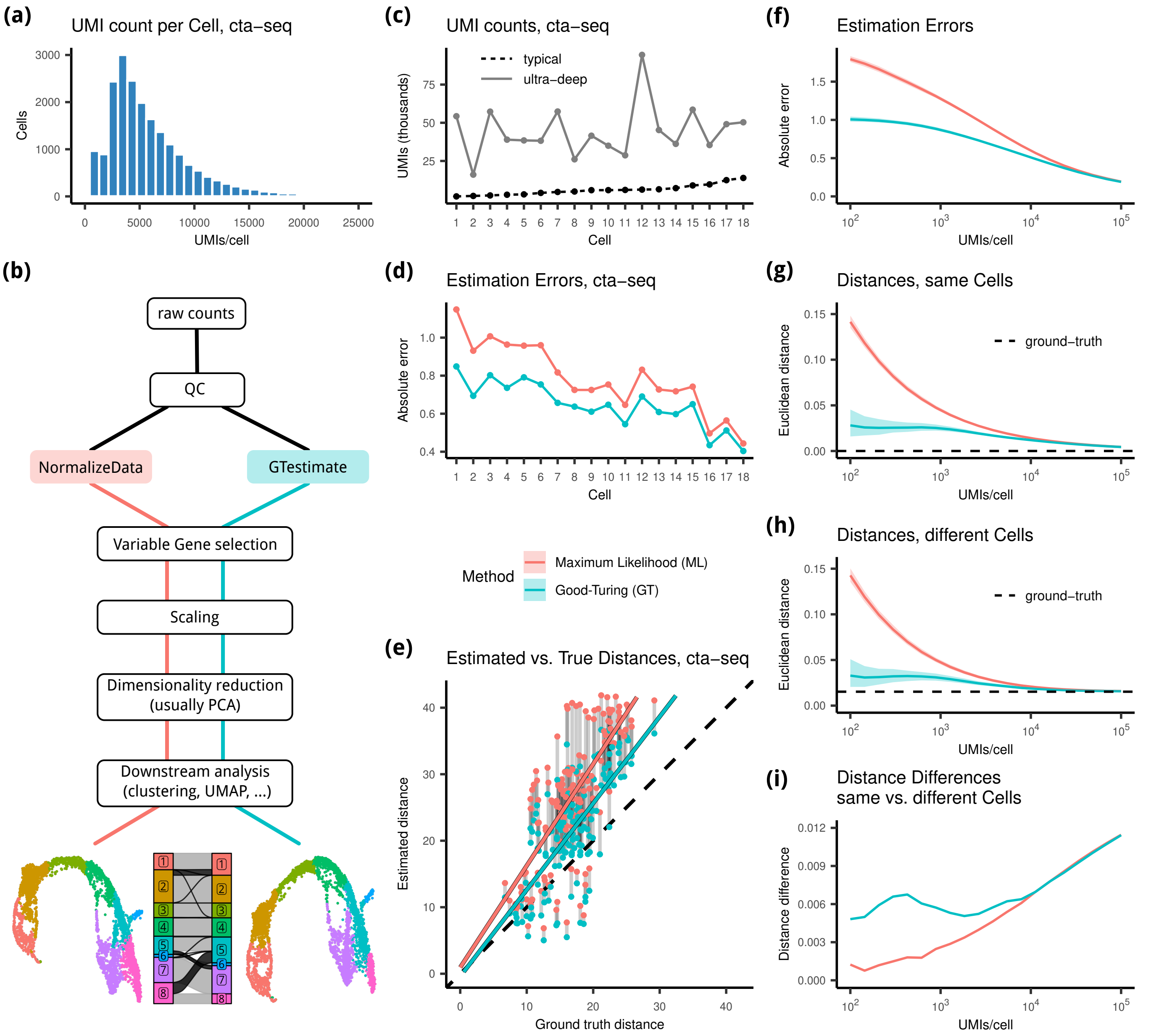

Figure 1: **(a)** Histogram of *UMIs/cell* for 17,653 cells in the cta-seq experiment before amplification. **(b)** Schema of a scRNA-seq analysis showing where *GTestimate* integrates into the workflow. **(c)** *UMIs/cell* for the 18 selected cells in the cta-seq experiment, before (*typical*) and after (*ultra-deep*) amplification. Cells ordered based on *UMIs/cell* in the *typical* cta-seq data. **(d)** Absolute error of the relative gene expression estimation in the cta-seq experiment. **(e)** Euclidean cell-cell distances in PCA-space in the cta-seq experiment. **(f)** Average absolute estimation error of the relative gene expression of a cell when subsampled to different *UMIs/cell*. **(g-h)** Mean Euclidean cell-cell distance in relative gene expression space, between two independent random samples of the same cell **(g)** between independent random samples of two different cells **(h)**. **(i)** Difference between the mean cell-cell distances in **(g)** and **(h)**. Colored ribbons in **(f,g,h)** represent the 5% – 95% quantile range.

where N_{c_g} denotes the number of genes with count c_g in the cell and $S()$ is a smoothing function following Gale and Sampson (1995) [17]. R1C4

GT adjusts the relative expression estimates of observed genes, particularly those with low counts, based R2C3 on the frequency of each count value in the cell. This even enables an estimate for the relative expression of unobserved genes (for further details see Suppl. Materials 1.1).

In this study, we first compare the performance of GT and ML on novel ultra-deep sequencing data, and then show how GT improves downstream results, by integrating it into standard scRNA-seq analysis workflows. To achieve this we developed *GTestimate*, a new scRNA-seq normalization method centered around GT. *GTestimate* is an easy-to-use R-package designed to seamlessly replace Seurat's *NormalizeData*.

Results

ultra-deep sequencing of single cells

Comparison between GT and ML requires ground-truth transcriptomic profiles of single cells. However, current simulation software cannot adequately emulate the complexity of scRNA-seq data and the choice of simulator may affect benchmarking results [18]. We therefore designed a cell targeted PCR-amplification strategy (cta-seq), which enabled us to sequence a small set of selected cells, from a *typical* sequencing run, a second time at a *ultra-deep* sequencing depth. This *ultra-deep* sequencing data contains an average of 23 million reads (44,511 *UMIs*, 7403 *genes*) per cell, a stark contrast to the average 16,965 reads (6,048 R1C5 *UMIs*, 2246 *genes*) for the same cells in the *typical* data (Figure S2). This represents a ~7.4 fold increase in *UMIs/cell* (Figure 1c) and a ~3.3 fold increase in *genes/cell* (Figure S3). We then used the relative gene expression levels of these *ultra-deep* profiles as the ground-truth for these cells.

Performance of GT and ML

Based on the cta-seq data we then evaluated GT and ML. When we applied GT and ML to the *typical* profiles and compared the results to the ground-truth, GT consistently showed a lower estimation error across all 18 cells, by ~17% on average (Figure 1d).

Relative gene expression profiles are the basis of most scRNA-seq analysis (Figure 1b), such as the calculation of cell-cell distances in PCA-space (often used as a measure for the similarity between two cells). We therefore also calculated cell-cell distances between the *typical* profiles, once based on GT and once based on ML, and compared the results to the cell-cell distances between the *ultra-deep* profiles. We observed a 36% reduction of the distance estimation error when using GT instead of ML (Figure 1e, Table S1).

Since *UMIs/cell* vary drastically (Figure 1a) we further assessed the performance of GT and ML at different *UMIs/cell*. We applied GT and ML to random subsamples of the cell with the highest *UMIs/cell*

in the *ultra-deep* cta-seq data (Cell 12, at 94,440 UMIs) and compared the estimates to the ground-truth expression profile of this cell. Similar to before (Figure 1d) the estimation error for both GT and ML decreased with increasing *UMIs/cell* and GT consistently showed a lower error than ML, especially at low *UMIs/cell* (Figure 1f).

Next, we assessed the impact of *UMIs/cell* on cell-cell distances. We first compared the mean distance between two random samples of the same cell (cell 12), both sampled to the same *UMIs/cell*. This distance was calculated in relative gene expression space and should approach zero for high *UMIs/cell*. However, ML led to grossly overestimated distances at small *UMIs/cell* (Figure 1g). The estimated distance after ML additionally showed strong correlation to the *UMIs/cell*, which is problematic as we assume that most of the observed variation in *UMIs/cell* is technical noise. In contrast, GT did not show correlation to the *UMIs/cell* and demonstrated lower distance estimation errors overall.

We then examined the distances between two distinct cells by also drawing random samples from the cell with the second highest *UMIs/cell* in the *ultra-deep* cta-seq data (cell 15, at 58,589 UMIs), which is of a different cell-type. We calculated the distances between the sampled profiles of cell 12 and cell 15 at varying *UMIs/cell*. We again saw large overestimation of the distances when using ML, while using GT strongly reduced this error. For high *UMIs/cell* the estimated distances converged to the true distance of 0.015 (Figure 1h).

When based on ML, the estimated distances between identical cells (Figure 1g) and distinct cells (Figure 1h) were almost the same for low *UMIs/cell*. This makes it very difficult to e.g. distinguish between cell-types. However, when we used GT as the basis for these distances we saw a much clearer separation between identical cells and cells of different cell-type, for cells with $< 10,000$ *UMIs/cell* (Figure 1i).

***GTestimate*'s impact on downstream results**

After showing GT's advantages for relative gene expression estimation and cell-cell distance estimation, we examined how our GT based normalization method *GTestimate* impacts downstream results. The difference between *GTestimate* and other *global-scaling normalization* methods is only in the estimator used, all other settings can be adjusted to be equivalent to e.g. scran's *computeSumFactors* or scanpy's *normalize_total*. At default settings *GTestimate* behaves identically to *NormalizeData*, including the same log-transformation. We therefore used *NormalizeData*, as a representative of ML based *global-scaling normalizations* for all following comparisons. However, we would expect similar results when comparing to other *global-scaling normalization* methods.

Direct comparison of normalized gene expression values across different normalization methods (e.g., R1C1
residual-based methods such as *SCTransform*) is often difficult due to varying scales and different data

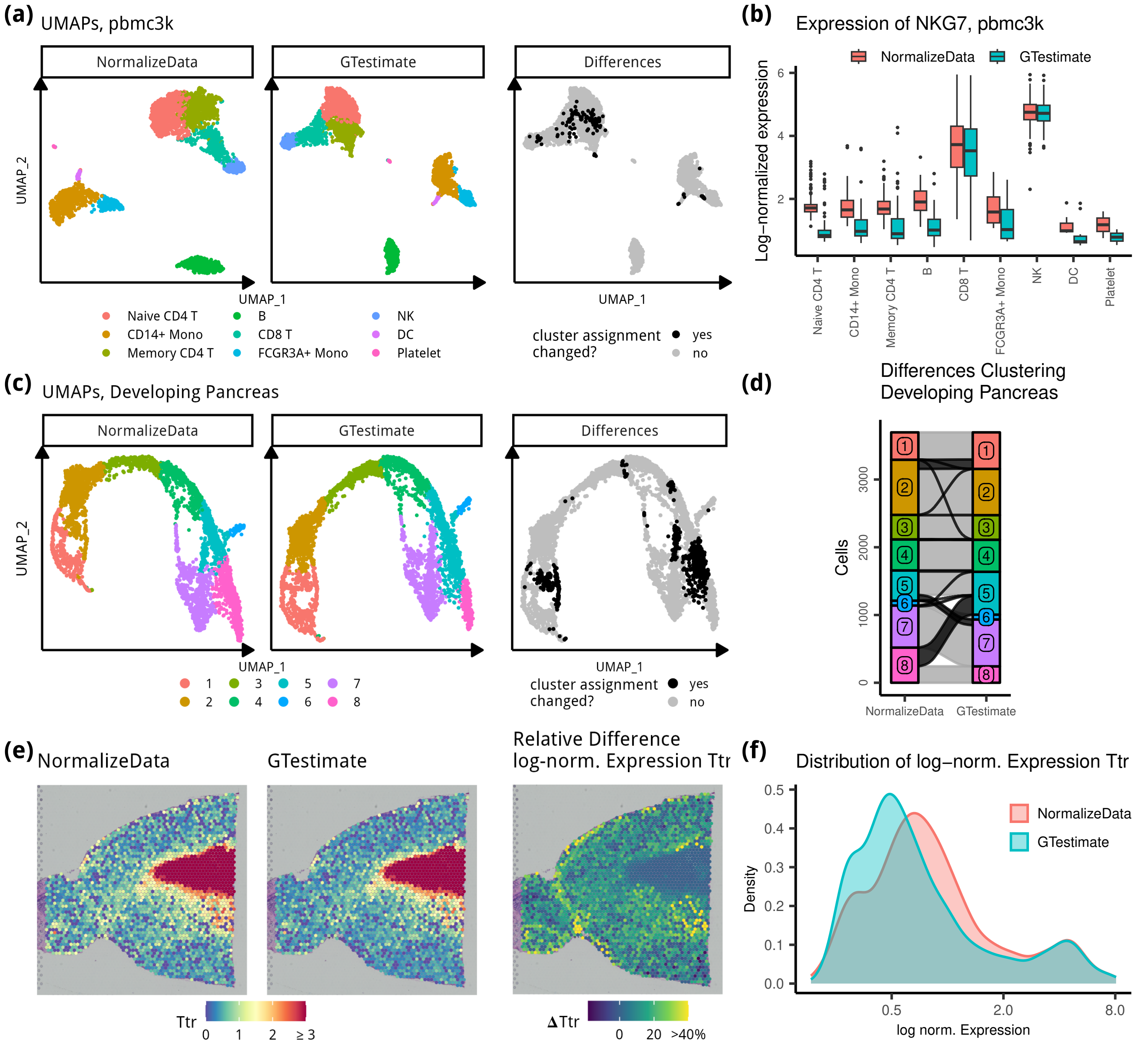

Figure 2: **pbmc3k: (a)** UMAPs based on *NormalizeData* and *GTestimate*, and UMAP highlighting differences in cluster assignment. **(b)** Boxplot showing log-normalized expression of *NKG7* per cell-type (zeroes not shown). **Developing Pancreas: (c)** UMAPs based on *NormalizeData* and *GTestimate*, and UMAP highlighting differences in cluster assignment. **(d)** Sankey diagram showing the differences in cluster assignment based on *NormalizeData* and *GTestimate*. **Spatial Transcriptomics: (e)** log-normalized gene expression of *Ttr* based on *NormalizeData* and *GTestimate* as well as percent difference in log-normalized expression of *Ttr* between *NormalizeData* and *GTestimate*. **(f)** Density plot showing the distribution of log-normalized gene expression values of *Ttr* for *NormalizeData* and *GTestimate*.

transformations. We therefore focus our initial comparison (Figure 2) on *NormalizeData*, representing global-scaling normalization methods. This choice aligns with recent findings indicating that global-scaling methods (followed by a log-transformation with pseudo-count and PCA) typically match or outperform more complex approaches [13]. However, we also provide a downstream clustering-based comparison including *SCTransform*.

We first assessed *GTestimate*'s impact on cell-type clustering by reanalyzing the pbmc3k data-set of peripheral blood mononuclear cells [19]. Here, normalization with *GTestimate* instead of *NormalizeData* resulted in 4.6% of cells being assigned to a different cluster (Figure 2a), mostly among the Naive CD4 T-cells, Memory CD4 T-cells and CD8 T-cells.

We additionally analyzed a developing pancreas data-set [20], characterized by more gradual cell-type transitions compared to the pbmc3k data-set. After normalization with *GTestimate* instead of *NormalizeData*, 14.6% of cells were assigned to a different cluster (Figure 2c,d).

While the correct classification of cells in both of these data-sets remains unknown, our results in Figure 1 suggest that *GTestimate* provides a better basis for this classification.

To examine the impact of *GTestimate* on the expression estimates of individual genes we considered the log-normalized expression of cell-type specific marker genes in the pbmc3k data-set. As an example we used *NKG7* a highly specific NK-cell and *CD8+* T-cell marker [21]. When using *GTestimate* instead of *NormalizeData*, the log-normalized expression of *NKG7* remained constant in NK-cells and *CD8+* T-cells, but was reduced in all other cell-types (Figure 2b). *GTestimate* therefore resulted in clearer separation of NK-cells and *CD8+* T-cells from other cell-types. We observed this for nearly all marker genes described in Seurat's pbmc3k tutorial (Figure S4). These differences may explain some of the observed changes in clustering.

We also applied *GTestimate* to the spot-wise normalization of a Spatial Transcriptomics data-set of the mouse brain [22]. In this data-set, normalization with *GTestimate* and *NormalizeData* resulted in 17 and 19 clusters respectively (Figure S5, Figure S6, Figure S7), we therefore refrained from any cluster based comparisons of *GTestimate* and *NormalizeData*. However, the spatial coordinates enabled examination of area specific marker genes, independent of the clustering. As an example we considered the log-normalized expression of the choroid plexus marker gene *Ttr* (Figure 2e). When using *GTestimate* we saw a reduction of the unspecific expression of *Ttr* for spots outside the choroid plexus. Here, *GTestimate* showed up to 50% reduction of the log-normalized expression, compared to *NormalizeData*, while expression estimates inside the choroid plexus remained constant (Figure 2e). This resulted in clearer separation of the choroid plexus spots from the surrounding tissue as shown by the distribution of expression values of *Ttr* (Figure 2f).

When we additionally considered the *UMIs/spot* (Figure S8), we saw a negative correlation between

the change in log-normalized expression of *Ttr* and *UMIs/spot*. This supports previous observations that *NormalizeData* overestimates the expression of *Ttr* in areas with low *UMIs/spot*. Whereas, *GTestimate* reduces this overestimation and improves the signal-to-noise ratio.

The data-sets shown in Figure 2a,c are widely used examples which highlight different aspects of scRNA-seq analysis. However, since these data-sets lack ground-truth cell-type annotations, we cannot conclusively evaluate clustering accuracy based on them alone. Although the clear differences observed when using *GTestimate* instead of *NormalizeData*, together with our earlier results (Figure 1), suggest improved relative gene-expression estimation with GT, this does not necessarily translate to better clustering performance. Direct benchmarking of clustering performance requires annotated data. R1C1 R1C7

To address this, we analyzed a recently published PBMC scRNA-seq data-set from Fu et al. [23], which includes experimentally annotated cell-types obtained via antibody-coated magnetic beads, providing a robust benchmark for clustering performance. We performed standard scRNA-seq analysis on this data-set, normalizing once with *GTestimate*, once with *NormalizeData* and once with *SCTransform*, followed by unsupervised clustering.

Here we also included *SCTransform*, as clustering is a downstream analysis step where differences in scaling and transformations become intrinsic properties of each normalization method. Consequently, the effects of these differences should be interpreted as advantages or disadvantages inherent to each approach.

We assessed clustering performance by calculating the Adjusted Rand Index (ARI) between the unsupervised clustering results and the provided cell-type annotations. The ARI ranges from 0 to 1, with 0 indicating no agreement and 1 indicating perfect agreement between two clustering. Because clustering outcomes strongly depend on the selected resolution parameter, we evaluated a broad range of resolutions from 0.1 to 1.5 (Figure S9).

Normalization with *GTestimate* produced higher ARI scores than *NormalizeData* at 14 of the 15 tested resolutions and outperformed *SCTransform* at 10 resolutions. Importantly, *GTestimate* also yielded the highest overall ARI (0.874), compared to 0.768 for *NormalizeData* and 0.822 for *SCTransform*. This superior maximum ARI is particularly relevant, as in practice the clustering resolution is routinely adjusted to optimize results. By this criterion, normalization with *GTestimate* provides the best clustering accuracy for this data-set.

Discussion

In summary, the estimation of relative gene expression is a central part of scRNA-seq data analysis, which has not received the same attention as other steps. We have shown that replacing the standard ML with GT improves relative gene expression estimation, without requiring expensive computations. By improving the

signal-to-noise ratio at this basic level, our new normalization method *GTestimate* can have large impact on downstream results.

In the validation we avoided potential issues with simulated data by employing a novel cell targeted PCR-amplification strategy to sequence the same cells at two vastly different *UMIs/cell*. This strategy may also be useful in other areas, such as the study of rare cell-types. Additionally, the resulting data-set may serve as a benchmark for other methods.

GTestimate is available as an open-source R-package (`https://www.github.com/Martin-Fahrenberger/GTestimate`) and works with all common scRNA-seq data-formats . While *GTestimate's* default behavior is designed to seamlessly replace *NormalizeData* it is also compatible with a wide variety of other workflows.

Materials and Methods

Implementation of *GTestimate*

The user-facing section of our *GTestimate* package was developed in R and handles input and output in the various supported data-formats. The core implementation of the Simple Good-Turing estimator is written in C++ and is heavily based on Aaron Lun's implementation for the edgeR R-package [24]. This core implementation includes the linear smoothing, which is necessary due to the sparsity of the frequencies of frequencies vector (i.e the frequency of the count values). It further includes a rescaling step which ensures that the estimated relative expression frequencies of all observed genes, plus the sum of probabilities of all unobserved genes (Suppl. Materials 1.1), add up to exactly one [17].

cta-seq experiment

In the cta-seq experiment we aimed to sequence a selected set of cells from a *typical* scRNA-seq library again at a *ultra-deep* sequencing depth. However, due to sequencing-saturation this quickly becomes prohibitively expensive. We therefore designed a PCR based cell targeted amplification strategy (cta-seq), to selectively amplify all transcripts from a small set of cells, through the use of primers specific to their cell-barcode. This is similar to the TAP-seq protocol [25], which uses gene-specific primers to amplify all transcripts of certain genes.

Sequencing cta-seq, *typical*

To ensure high quality input material we used leftover cDNA from a previously sequenced sample [26], which had shown high *UMIs/cell* and *genes/cell*. The sample was taken out of -20C storage and prepared for Illumina sequencing at the Vienna Biocenter Next Generation Sequencing facility using 10X Dual Index Kit TT. We then split the resulting sequencing library into two aliquots and stored the second halve again

at -20C. The first halve was sequenced on a Illumina NovaSeq S4 in paired-end mode with 2x150bp read length and 400 million reads.

Sequencing cta-seq, *ultra-deep*

Based on the results from the *typical* sequencing run we selected 18 cells of interest for the cta-seq experiment (see below). For these 18 cells we designed PCR primers specific to their cell-barcodes. We used the second aliquote of the previously prepared sequencing library and split it further into 18 individual reactions, one R1C6 for each targeted cell. We then performed three rounds of PCR amplification with the respective primers using Amplitaq Gold 360 MM (ThermoFisher, cat.: 4398886) supplemented with EvaGreen dye (Biotium, cat.: 31000). We used the following programs in a total volume of 50μl.. PCR1: 1. 95C, 10min; 2. 62C, 30s; 3. 72C, 2min.; 4. Return to 2. x2; 5. 95C, 25s; 6. 62C, 30s; 7. 72C, 2min, fluorescence measurement; 8. 72C, 15s; 9. return to 5. x16. PCR2: 1. 95C, 10min; 2. 62C, 30s; 3. 72C, 2min.; 4. Return to 2. x2; 5. 95C, 25s; 6. 62C, 30s; 7. 72C, 2min, fluorescence measurement; 8. 72C, 15s; 9. return to 5. x16. PCR3: 1. 95C, 10min; 2. 67C, 30s; 3. 72C, 2min.; 4. Return to 2. x2; 5. 95C, 25s; 6. 67C, 30s; 7. 72C, 2min, fluorescence measurement; 8. 72C, 15s; 9. return to 5. x8. Reactions were stopped in step 8 according to fluorescent measurements in log phase. Reaction input in PCRs 2 and 3 were 0.5 μl of the previous reaction. Resulting reactions were purified, and pooled for Illumina sequencing on a NovaSeq S4 in paired-end mode with 2x150bp read length and 400 million reads. The primer sequences used can be found in Table 1, PCR1 primers were designed with varying length to achieve similar melting temperatures.

Data Analysis

All data analysis was performed in R (v4.3.1) using Seurat (v5.0.0) functions at default settings unless stated otherwise.

Data analysis, cta-seq *typical* depth

We first processed the *typical* depth sequencing data using CellRanger (v7.1.0), this resulted in 20,214 cells. During cell QC we then removed all cells expressing $\leq$ 1000 or $\geq$ 5000 genes as well as cells with $\geq$ 8% mitochondrial reads, with 17,653 cells remaining. We then normalized with Seurats *NormalizeData*, selected the top 2000 most variable genes and performed gene-wise z-score scaling. Next we applied PCA and performed unsupervised clustering of cells using the Louvain algorithm [27](resolution = 0.1), based on the first 50 principal components (PCs). This resulted in four cell-type clusters, the smallest cluster (with only 504 cells) was excluded from the subsequent analysis.

From the remaining 17,149 cells we selected 18 cells for targeted amplification, six cells from each of the three remaining clusters. To select a diverse set of cells from each cluster we used the following:

1. We identified the two nearest neighbors for each cell (in PCA space).
2. We excluded cells for which at least one nearest neighbor belonged to a different cluster.
3. For the remaining 16,295 cells, we computed the #UMI-rank, from the number of observed UMIs per cell (ties were broken randomly).
4. Similarly, we computed the $\frac{\#\text{UMI}}{\#\text{Genes}}$-rank based on the ratio of the number of observed UMIs and the number of observed genes in the cell (ties were broken randomly).
5. Subsequently, we calculated the diversity of each cell and it's neighbors as the area of the induced triangle of the cell and its neighbors in a #UMI-rank x $\frac{\#\text{UMI}}{\#\text{Genes}}$-rank plot. The six cells from the two most diverse neighborhoods (i.e. largest triangle area) were selected for amplification.

These steps were designed to cover a diverse set of cells for which the various experimental steps had varying efficiencies. The selection of triplets from the same neighborhoods provided groups of cells with similar gene expression patterns, while the number of UMIs and the number of observed genes were used as proxies for the mRNA capture efficiencies and the health of the isolated cells.

Data analysis, cta-seq *ultra-deep*

The sequencing data from the *ultra-deep* sequencing run were processed using CellRanger (v7.1.0).

However, due to the high number of PCR cycles during amplification, and the resulting high number of reads for the 18 selected cells, CellRanger's UMI correction approach was no longer sufficient. Manual inspection of the reads showed that errors in the UMI sequences had inflated the number of unique reads.

This was further exacerbated by a faulty implementation of the UMI-correction approach in the CellRanger software by 10X Genomics. CellRanger erroneously corrects UMIs containing sequencing errors towards other UMIs that also contain sequencing errors. E.g. If we have 3 UMIs: AAAA with 10 reads, AAAT with 2 reads and AATT with 1 read, AATT would be corrected towards AAAT (Hamming Distance 1) and stay as AAAT, eventhough the original 2 AAAT reads would be corrected to AAAA in the same step. We have reported this issue to 10X Genomics on 13th of July 2023, 10X Genomics acknowledge the issue on 14th of July 2023. The issue remains unresolved in CellRanger 7.2.0 (released on the 10th of November, 2023).

To circumvent these issues we extracted the relevant information for each read (count, ensemble gene id, cell-barcode, uncorrected UMI and CellRanger corrected UMI) from the possorted_genome_bam.bam as provided by CellRanger and replicated CellRanger's read counting workflow in R. As a sanity-check we first used the CellRanger corrected UMIs and achieved the exact same count-matrix as CellRanger. We then used the raw UMIs instead of the CellRanger corrected UMIs, implemented the UMI-tools directional UMI correction approach [28] in R and applied it to correct the UMIs for the 18 selected cells, we then counted

again. The resulting count-matrix showed differences for 28% of the non-zero entries when compared to the CellRanger results. We used these improved counts for the *ultra-deep* profiles in all further analysis.

Comparison of GT and ML using cta-seq

To evaluate the performance of GT and ML based on the cta-seq data-set we estimated the relative gene expression for the 18 selected cells by applying both estimators to the *typical* transcriptomic profiles.

The relative gene expression for the ground-truth *ultra-deep* profiles was estimated with ML. We chose ML to be conservative regarding the performance of GT and since the overestimation due to unobserved genes should be small for the *ultra-deep* profiles Figure S10.

Relative gene expression estimation

We calculated the absolute estimation error for the relative gene expression of the 18 cells by comparing the estimation results of GT and ML based on the *typical* transcriptomic profiles to the ground-truth relative gene expression of the *ultra-deep* profiles. We consider the relative gene expression estimation error of a cell to be the sum of the individual relative gene expression estimation errors in the cell.

Cell-cell distances

The pairwise Euclidean distances between the 18 cells were calculated in PCA space (as is common for cell-cell distances in scRNA-seq). However, to keep the necessary projections similar to a regular scRNA-seq analysis this space could not simply be constructed based only on the 18 selected cells.

Instead we calculated the projections based on 17,653 cells in the *typical* sequencing run. After normalization there are three pre-processing steps which all depend on the context of a full data-set; Variable gene selection, gene-wise z-score scaling and PCA.

To keep these steps identical for both the GT and ML profiles of the *typical* sequenced cells, as well as the *ultra-deep* profiles we performed them using customized functions. We used the same list of variable genes (calculated based on all 17,653 cells) for the analysis of all profiles. We then scaled the genes in all profiles using the mean and standard deviation of genes calculate based on the full 17,653 cells. Finally we projected all profiles into the same 50 dimensional PCA-space calculated from the full 17,653 cells.

In this PCA-space we calculated the pairwise distances between the ML profiles, between the GT profiles as well as between the ground-truth *ultra-deep* profiles. We then compared the resulting non-zero distances based on GT and ML to the ground-truth *ultra-deep* distances.

Comparison of GT and ML at different *UMIs/cell*

When analyzing the impact of *UMIs/cell* on the estimation performance we used the cell with the highest number of UMIs after amplification (cell 12, cell-barcode TCTCTGGGTGTGCTTA) and the cell with the second highest number of UMIs after amplification (cell 15, cell-barcode GGCTTTCGTGTGTCGC).

We generated 1000 randomly sampled profiles at each *UMIs/cell* level by drawing genes from the *ultra-deep* count-vector, weighted by count and with replacement. The 20 *UMIs/cell* levels at which we sampled were chosen equidistant in log10-space from 100 to 100,000 (i.e. 100, 143, 206, 297, 428, 615, 885, 1274, 1832, 2636, 3792, 5455, 7847, 11288, 16237, 23357, 33598, 48329, 69519, 100000 *UMIs/cell*). We then applied GT and ML respectively to these sampled profiles to estimate their relative gene expression.

Relative gene expression estimation

To asses the relative gene expression estimation performance of GT and ML we compared their estimates for each sampled profile from cell 12 to the relative gene expression of the full *ultra-deep* profile of cell 12, and calculated the absolute error.

Cell-cell distance estimation

To asses cell-cell distance estimation performance we calculated the Euclidean distances between the relative gene expression profiles of pairs of sampled profiles (either from cell 12 twice or from cell 12 and cell 15) based on GT and ML. We calculated the true distance based on the full *ultra-deep* profiles.

Downstream analysis

Data analysis, pbmc3k

The pbmc3k data-set was downloaded from 10X Genomics [19] and processed following Seurat's "Guided Clustering Tutorial" [29]. In short:

During QC we filtered out genes expressed in less than 3 cells, and cells with less than 200 expressed genes. We then filtered out cells with $> 5\%$ mitochondrial reads and finally we removed all cells expressing more than 2,500 genes.

During preprocessing cells were normalized using either Seurat's *NormalizeData* or *GTestimate* at default settings. For both normalization methods individually, we then identified variable genes and z-score scaled the data, followed by calculation of the top 10 PCs. Based on these PCs we then constructed the neighborhood graphs and performed unsupervised Louvain clustering (resolution = 0.5). Finally we calculated the UMAP for both conditions and annotated clusters based on marker gene expression, following the Seurat tutorial.

Data analysis, developing pancreas

The pancreas endocrinogenesis day15 dataset was downloaded [30] and imported into R to be processed using Seurat. We only used the spliced counts and normalized them using *GTestimate* and *NormalizeData*; from there on all following steps were performed identically for the two approaches.

First we identified variable genes and performed gene-wise z-score scaling, followed by calculation of the top 50 PCs. Based on the PCs we constructed the neighborhood graph and performed unsupervised Louvain clustering (resolution = 0.4). Finally we calculated the UMAP.

We manually adjusted the cluster numbering (and thereby their color) for Fig. 2c and Fig. 2d. to have consistent cluster-colors from left to right.

Data analysis, Spatial Transcriptomics

The stxBrain data-set of sagital mouse brain slices from 10X Genomics was downloaded using the SeuratData R-package. In our analysis we focused on the anterior1 slice of the data-set following Seurat's "Analysis of spatial datasets (Sequencing-based)" vignette [31].

Our analysis differs from the vignette only in the normalization methods used. While the vignette uses *sctransform*[32] for spot-wise normalization we instead used *NormalizeData* and *GTestimate*. Direct comparison of GT and ML to *SCTransform* on the basis of relative gene expression is not possible, since *SCTransform* does not calculate relative gene expression levels. Normalization was followed by variable gene selection and gene-wise scaling. We then calculated the first 30 PCs and used them to construct the neighborhood graph, perform unsupervised Louvain clustering and calculate the UMAP.

Data analysis, experimentally annotated PBMCs (Liu data-set)

The Liu data-set was downloaded and imported into R to be processed using Seurat. We used the purified R1C7
version of the data-set, which includes an additional filtering step to ensure correct cell-type assignments.

For our *GTestimate* and *NormalizeData* analyses we first normalized the data using the respective method at default settings, we then identified the 2,000 most variable genes and performed gene-wise z-score scaling. For our *SCTransform* based analysis we simply applied *SCTransform* at it's default settings, as it is supposed to replace all three of these steps.

From here the remaining steps were identical for the three analyses: We first calculated the top 30 PCs (we chose 30 PCs to be inline with the original analysis by Fu et al. [23] performed as part of their cell filtering step) and then constructed the neighborhood-graph and performed unsupervised Louvain clustering. Louvain clustering was repeated at 15 different resolutions from 0.1 to 1.5 in steps of 0.1.

At each resolution we calculated the ARI between the experimentally annotated ground-truth cell-types

and the unsupervised clustering results.

Availability of supporting source code and requirements

1. Project name: GTestimate
2. Project home page: https://github.com/Martin-Fahrenberger/GTestimate
3. Operating system(s): Platform independent
4. Programming language: R, C++
5. Other Requirements: devtools, sparseMatrixStats
6. License: GPL3

GTestimate is available as an open-source R-package on github (https://www.github.com/Martin-Fahrenberger/GTestimate), RRID: SCR_026562, biotoolsID: biotools:gtestimate. All code for the analysis in this paper, from raw-data to figures, is available on github (https://www.github.com/Martin-Fahrenberger/GTestimate-Paper).

Data Availability

Processed cta-seq data, such as count-matrices, are available via GEO (https://www.ncbi.nlm.nih.gov/geo/), accession number GSE268930. Due to patient privacy concerns raw sequencing data will be made available through controlled access at the European Genome-Phenome Archive (EGA) upon publication.

List of Abbreviations

cta-seq: cell targeted PCR-amplification followed by sequencing; GT: Good-Turing estimator; ML: Maximum Likelihood estimator; PC: principal component; scRNA-seq: single-cell RNA-sequencing; ARI: Adjusted Rand Index

Competing interests

The authors declare that they have no competing interests.

Funding

This work was supported by the network grant of the European Commission H2020-MSCA-ITN-2017-765104 MATURE-NK to AvH; MF was a fellow in the project. MF was further supported by the Austrian Science Fund (FWF) project number F78 to AvH.

Authors' contributions

MF and AvH conceived this project, CE and MF developed cta-seq, JK provided the cDNA samples, CE performed the cta-seq wet-lab experiments in the lab of JK, MF implemented GTestimate and analyzed the data. MF wrote the manuscript with input from CE and AvH. All authors read and approved the final version of the manuscript.

Acknowledgments

We thank Oliver L. Eichmüller for the original cDNA-library used in the cta-seq experiment and for his feedback during discussions. We thank all members of CIBIV for their valuable feedback throughout this project. We also thank Thomas Grentzinger from the Vienna BioCenter Core Facilities GmbH (VBCF) Next Generation Sequencing Unit for consultation and sequencing.

References

[1] Aviv Regev, Sarah A Teichmann, Eric S Lander, Ido Amit, Christophe Benoist, Ewan Birney, Bernd Bodenmiller, Peter Campbell, Piero Carninci, Menna Clatworthy, et al. The human cell atlas. *elife*, 6: e27041, 2017.

[2] Jeffrey A Farrell, Yiqun Wang, Samantha J Riesenfeld, Karthik Shekhar, Aviv Regev, and Alexander F Schier. Single-cell reconstruction of developmental trajectories during zebrafish embryogenesis. *Science*, 360(6392):eaar3131, 2018.

[3] Jihwan Park, Rojesh Shrestha, Chengxiang Qiu, Ayano Kondo, Shizheng Huang, Max Werth, Mingyao Li, Jonathan Barasch, and Katalin Suszták. Single-cell transcriptomics of the mouse kidney reveals potential cellular targets of kidney disease. *Science*, 360(6390):758–763, 2018.

[4] Evan Z Macosko, Anindita Basu, Rahul Satija, James Nemesh, Karthik Shekhar, Melissa Goldman, Itay Tirosh, Allison R Bialas, Nolan Kamitaki, Emily M Martersteck, et al. Highly parallel genome-wide expression profiling of individual cells using nanoliter droplets. *Cell*, 161(5):1202–1214, 2015.

[5] Grace XY Zheng, Jessica M Terry, Phillip Belgrader, Paul Ryvkin, Zachary W Bent, Ryan Wilson, Solongo B Ziraldo, Tobias D Wheeler, Geoff P McDermott, Junjie Zhu, et al. Massively parallel digital transcriptional profiling of single cells. *Nature communications*, 8(1):14049, 2017.

[6] 10X Genomics. Technical Note - Chromium Single Cell 3' v3: Reagent, Workflow & Software Updates, 25 Febuary, 2019. Document Number CG000201, Rev A.

[7] Allon M Klein, Linas Mazutis, Ilke Akartuna, Naren Tallapragada, Adrian Veres, Victor Li, Leonid Peshkin, David A Weitz, and Marc W Kirschner. Droplet barcoding for single-cell transcriptomics applied to embryonic stem cells. *Cell*, 161(5):1187–1201, 2015.

[8] Catalina A Vallejos, Davide Risso, Antonio Scialdone, Sandrine Dudoit, and John C Marioni. Normalizing single-cell rna sequencing data: challenges and opportunities. *Nature methods*, 14(6):565–571, 2017.

[9] Andrew Butler, Paul Hoffman, Peter Smibert, Efthymia Papalexi, and Rahul Satija. Integrating single-cell transcriptomic data across different conditions, technologies, and species. *Nature biotechnology*, 36 (5):411–420, 2018.

[10] Aaron TL Lun, Davis J McCarthy, and John C Marioni. A step-by-step workflow for low-level analysis of single-cell rna-seq data with bioconductor. *F1000Research*, 5, 2016.

[11] Aaron T L Lun, Karsten Bach, and John C Marioni. Pooling across cells to normalize single-cell rna sequencing data with many zero counts. *Genome biology*, 17(1):1–14, 2016.

[12] F Alexander Wolf, Philipp Angerer, and Fabian J Theis. Scanpy: large-scale single-cell gene expression data analysis. *Genome biology*, 19:1–5, 2018.

[13] Constantin Ahlmann-Eltze and Wolfgang Huber. Comparison of transformations for single-cell rna-seq data. *Nature Methods*, pages 1–8, 2023.

[14] Ronald A Fisher. On the mathematical foundations of theoretical statistics. *Philosophical transactions of the Royal Society of London. Series A, containing papers of a mathematical or physical character*, 222(594-604):309–368, 1922.

[15] Ehud Shapiro, Tamir Biezuner, and Sten Linnarsson. Single-cell sequencing-based technologies will revolutionize whole-organism science. *Nature Reviews Genetics*, 14(9):618–630, 2013.

[16] Irving J Good. The population frequencies of species and the estimation of population parameters. *Biometrika*, 40(3-4):237–264, 1953.

[17] William A Gale and Geoffrey Sampson. Good-turing frequency estimation without tears. *Journal of quantitative linguistics*, 2(3):217–237, 1995.

[18] Helena L Crowell, Sarah X Morillo Leonardo, Charlotte Soneson, and Mark D Robinson. The shaky foundations of simulating single-cell rna sequencing data. *Genome Biology*, 24(1):1–19, 2023.

[19] 10X Genomics. 3k PBMCs from a Healthy Donor, Single Cell Gene Expression Dataset by Cell Ranger 1.1.0, 26 May, 2016.

[20] Aimée Bastidas-Ponce, Sophie Tritschler, Leander Dony, Katharina Scheibner, Marta Tarquis-Medina, Ciro Salinno, Silvia Schirge, Ingo Burtscher, Anika Böttcher, Fabian J Theis, et al. Comprehensive single cell mrna profiling reveals a detailed roadmap for pancreatic endocrinogenesis. *Development*, 146 (12):dev173849, 2019.

[21] Martin A Turman, Toshio Yabe, Cynthia McSherry, Fritz H Bach, and Jeffrey P Houchins. Characterization of a novel gene (nkg7) on human chromosome 19 that is expressed in natural killer cells and t cells. *Human immunology*, 36(1):34–40, 1993.

[22] 10X Genomics. Mouse Brain Serial Section 1 (Sagittal-Anterior), Spatial Gene Expression Dataset by Space Ranger 1.0.0, 02 December, 2019.

[23] Qiqing Fu, Chenyu Dong, Yunhe Liu, Xiaoqiong Xia, Gang Liu, Fan Zhong, and Lei Liu. A comparison of scrna-seq annotation methods based on experimentally labeled immune cell subtype dataset. *Briefings in Bioinformatics*, 25(5):bbae392, 2024.

[24] Yunshun Chen, Lizhong Chen, Aaron TL Lun, Pedro L Baldoni, and Gordon K Smyth. edger 4.0: powerful differential analysis of sequencing data with expanded functionality and improved support for small counts and larger datasets. *bioRxiv*, pages 2024–01, 2024.

[25] Daniel Schraivogel, Andreas R Gschwind, Jennifer H Milbank, Daniel R Leonce, Petra Jakob, Lukas Mathur, Jan O Korbel, Christoph A Merten, Lars Velten, and Lars M Steinmetz. Targeted perturb-seq enables genome-scale genetic screens in single cells. *Nature methods*, 17(6):629–635, 2020.

[26] Oliver L Eichmüller, Nina S Corsini, Ábel Vértesy, Ilaria Morassut, Theresa Scholl, Victoria-Elisabeth Gruber, Angela M Peer, Julia Chu, Maria Novatchkova, Johannes A Hainfellner, et al. Amplification of human interneuron progenitors promotes brain tumors and neurological defects. *Science*, 375(6579): eabf5546, 2022.

[27] Vincent D Blondel, Jean-Loup Guillaume, Renaud Lambiotte, and Etienne Lefebvre. Fast unfolding of communities in large networks. *Journal of statistical mechanics: theory and experiment*, 2008(10): P10008, 2008.

[28] Tom Smith, Andreas Heger, and Ian Sudbery. Umi-tools: modeling sequencing errors in unique molecular identifiers to improve quantification accuracy. *Genome research*, 27(3):491–499, 2017.

[29] Satija-Lab. Seurat - guided clustering tutorial, 2023. URL https://satijalab.org/seurat/articles/pbmc3k_tutorial. Accessed on 13.12.2023.

[30] Theis Lab. scvelo - github page, 2021. URL https://github.com/theislab/scvelo_notebooks/raw/master/data/Pancreas/endocrinogenesis_day15.h5ad. Accessed on 13.12.2023.

[31] Satija-Lab. Analysis, visualization, and integration of spatial datasets with seurat, 2023. URL https://satijalab.org/seurat/articles/spatial_vignette. Accessed on 13.12.2023.

[32] Saket Choudhary and Rahul Satija. Comparison and evaluation of statistical error models for scrna-seq. *Genome biology*, 23(1):27, 2022.

1 Supplementary Materials

1.1 The Missing Mass

Besides improving the relative expression estimates of observed genes, GT can also estimate the sum of the relative frequencies of all unobserved genes. This can be viewed as the probability p_0 that a next hypothetical UMI would be of a currently unobserved gene. We have therefore termed p_0 the missing-mass of the relative gene expression distribution.

The missing-mass for each cell is estimated from the number of genes with a UMI count of one (N_1) and the sum of all counts ($\sum_g c_g$) as has previously been discussed [16, 17].

$$\widehat{p_0} = \frac{N_1}{\sum_g c_g} \tag{S1}$$

When applied to a Seurat or SingleCellExperiment object in R *GTestimate* saves the estimated $\widehat{p_0}$ for each cell into a meta-data vector called "missing_mass".

The Simple Good-Turing estimator scales the relative frequencies (including p_0) to ensure

$$\sum_g \widehat{f_g}^{GT} + \widehat{p_0} = 1 \tag{S2}$$

for each cell.

Equation S1 provides insight into the amount of information present for each cell, which may warrant further study. E.g. the missing-mass in the cta-seq experiment is substantially reduced after cell targeted amplification of reads (Fig. S10).

Due to the typically low *UMIs/cell*, this missing mass of a cell in scRNA-seq can be quite substantial (Fig. S11).

1.2 Supplementary Tables

Method	Slope	Sum of absolute Residuals	Intercept	Sum of absolute Errors
ML	1.529	1511.317	0.955	3258.049
GT	1.302	1263.276	-0.408	2093.645

Table S1: Characteristics of the regression line of the estimated vs. ground-truth distances for the cta-seq data (Fig. 1d).

1.3 Supplementary Figures

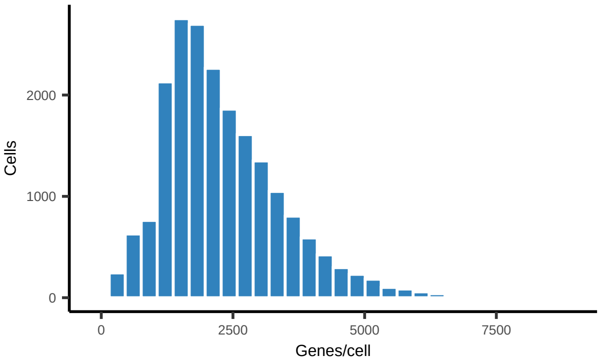

Figure S1: Histogram showing the number of observed genes per cell for the 17,653 cells in the cta-seq sample before amplification (*typical*).

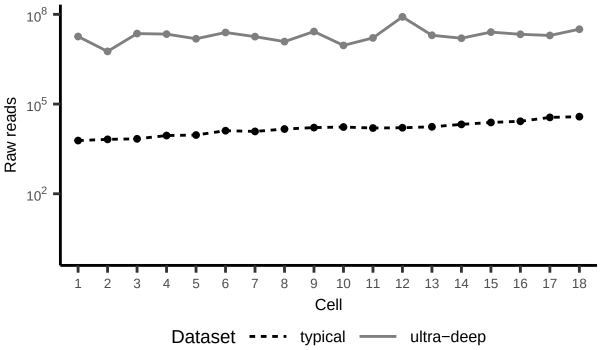

Figure S2: Raw read counts per cell before (*typical*) and after (*ultra-deep*) amplification for the 18 selected cells in the cta-seq experiment.

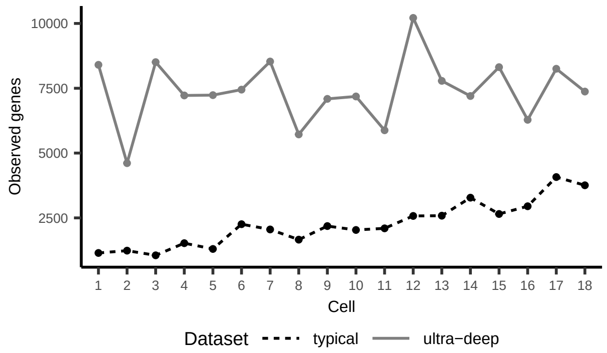

Figure S3: Number of observed genes before (*typical*) and after (*ultra-deep*) amplification for the 18 selected cells in the cta-seq experiment.

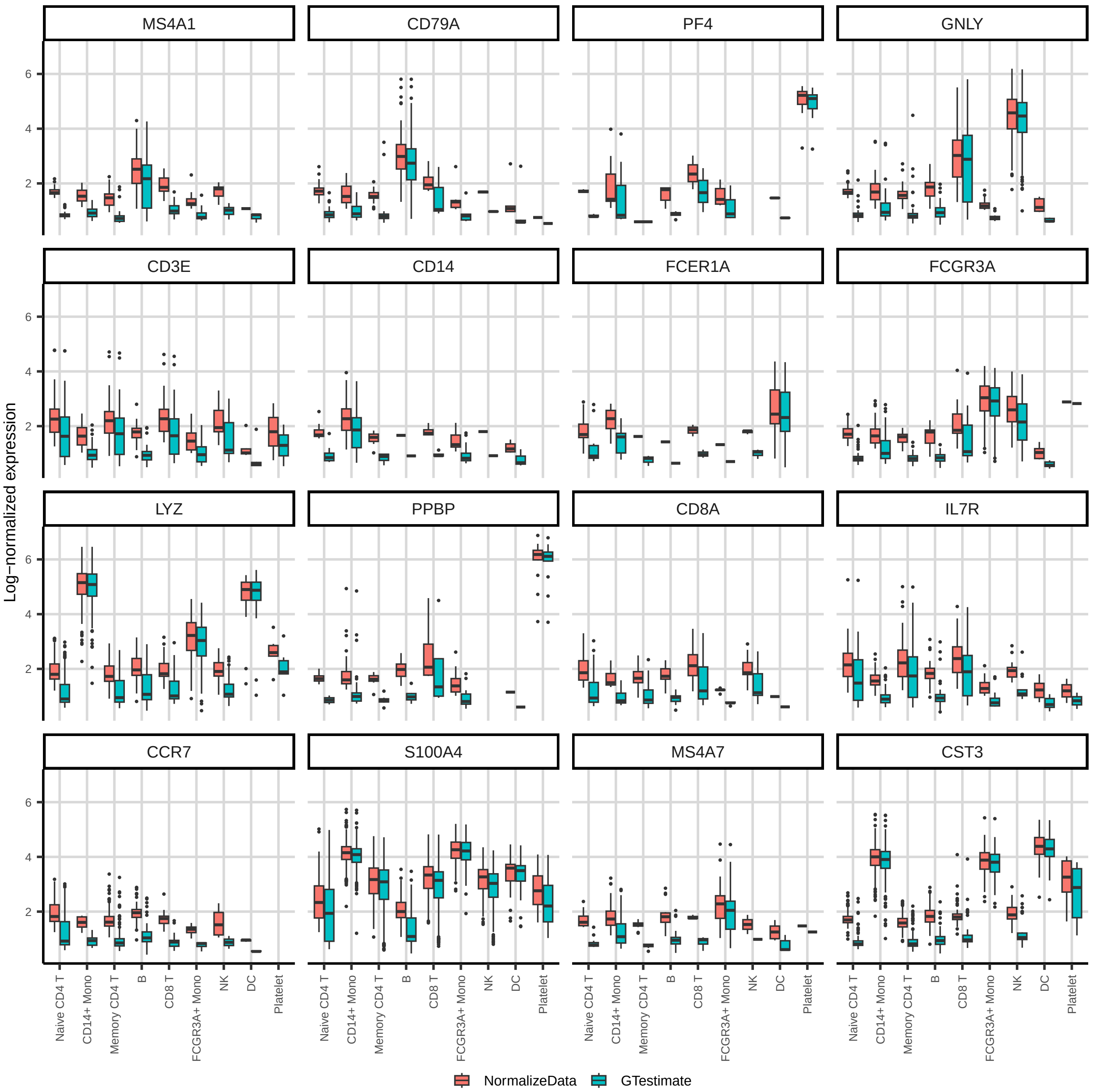

Figure S4: Log-normalized expression of all cell-type markers described in Seurat's pbmc3k tutorial (zeroes not shown).

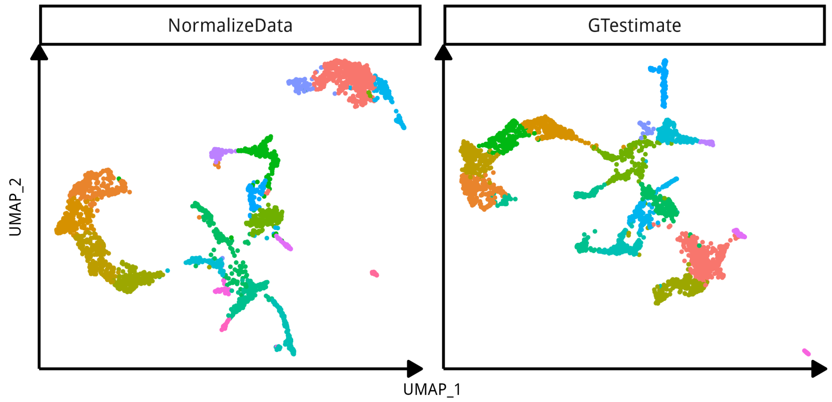

Figure S5: UMAPs visualizing the clustering of Spatial Transcriptomics spots, based on *NormalizeData* **(left)** and *GTestimate* **(right)** for the mouse brain Spatial Transcriptomics data-set.

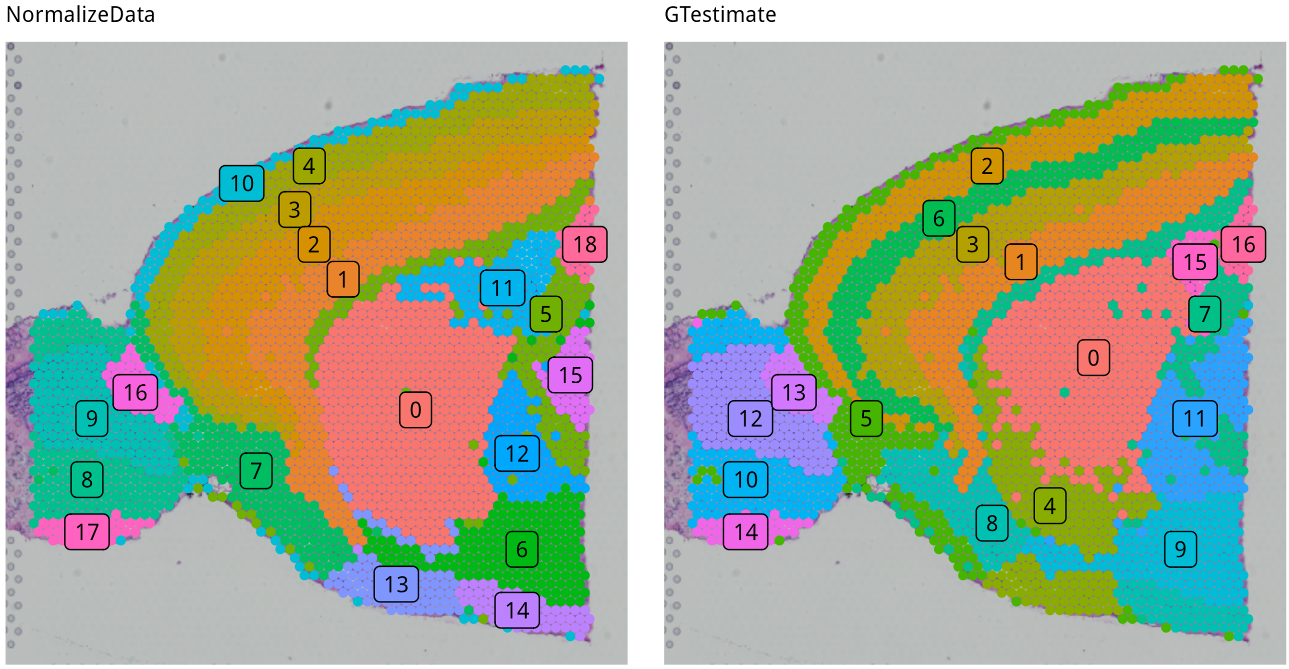

Figure S6: Visualization of the different clusters based on *NormalizeData* **(left)** and *GTestimate* **(right)** for the mouse brain Spatial Transcriptomics data-set.

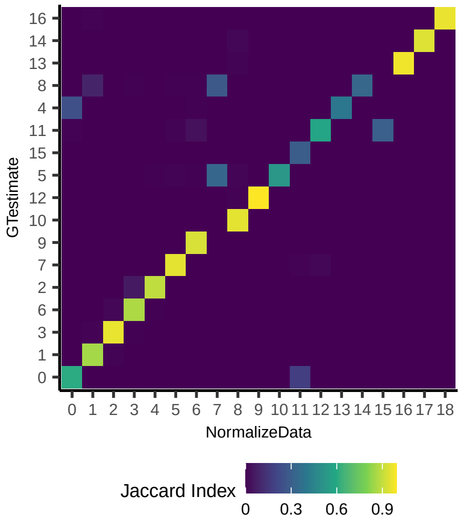

Figure S7: Similarity of the clusters based on *NormalizeData* and *GTestimate* as represented by the Jaccard Index. Clusters on the y-axis have been rearrange to maximize diagonal entries using the Hungarian Algorithm.

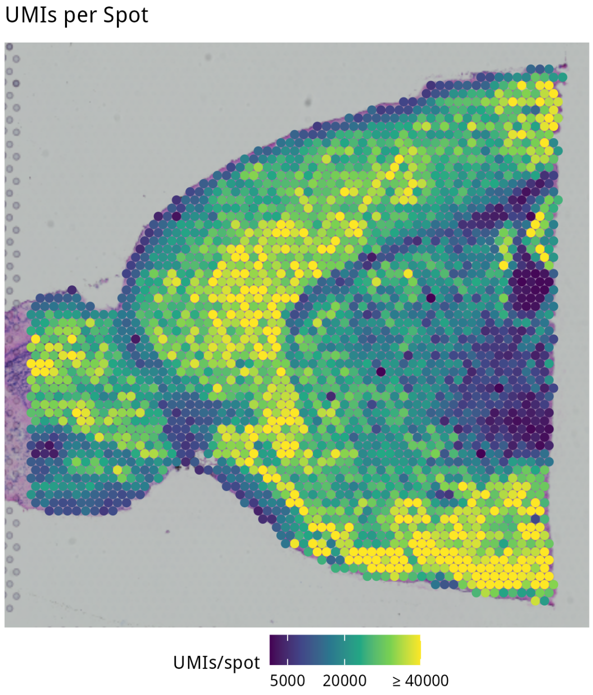

Figure S8: *UMIs/spot* in the Spatial Transcriptomics mouse brain data-set.

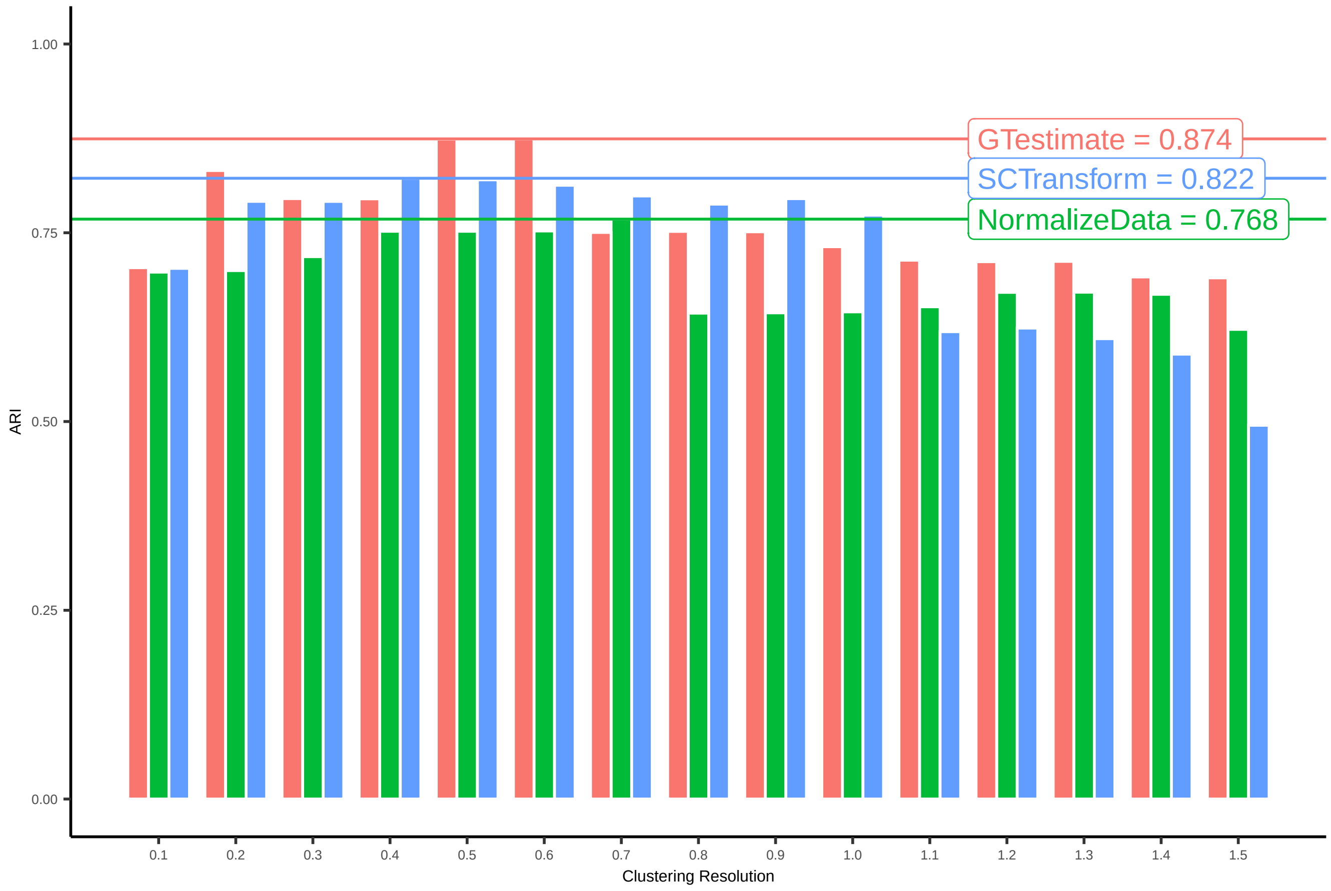

Figure S9: Adjusted Rand Index (ARI) comparing unsupervised clustering results (Louvian algorithm), to the experimentally annotated cell-types in the liu data-set. Clustering was performed after normalizing with either GTestimate, NormalizeData or SCTransform and repeated for different clustering resolutions. The maximum ARI for each normalization method is indicated and labeled.

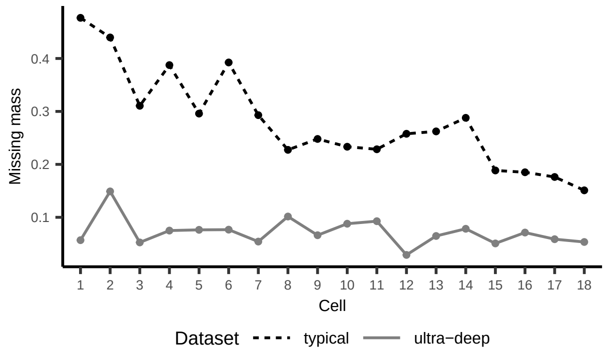

Figure S10: Missing mass before (*typical*) and after (*ultra-deep*) amplification for the 18 selected cells in the cta-seq experiment (see Suppl. Materials 1.1).

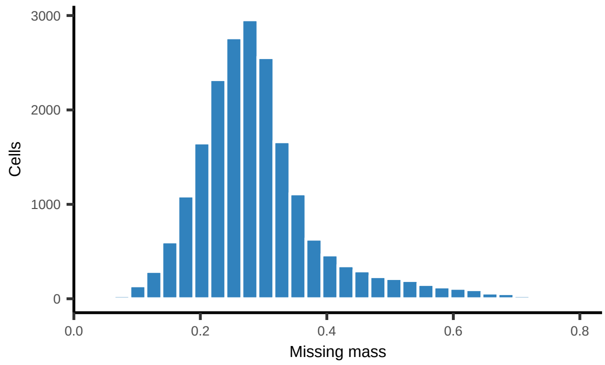

Figure S11: Histogram showing *GTestimate*'s missing mass estimates per cell for the 17,653 cells in the cta-seq sample before amplification (*typical*).

Figure 2

Click here to access/download;Figure;fig_2_new.pdf

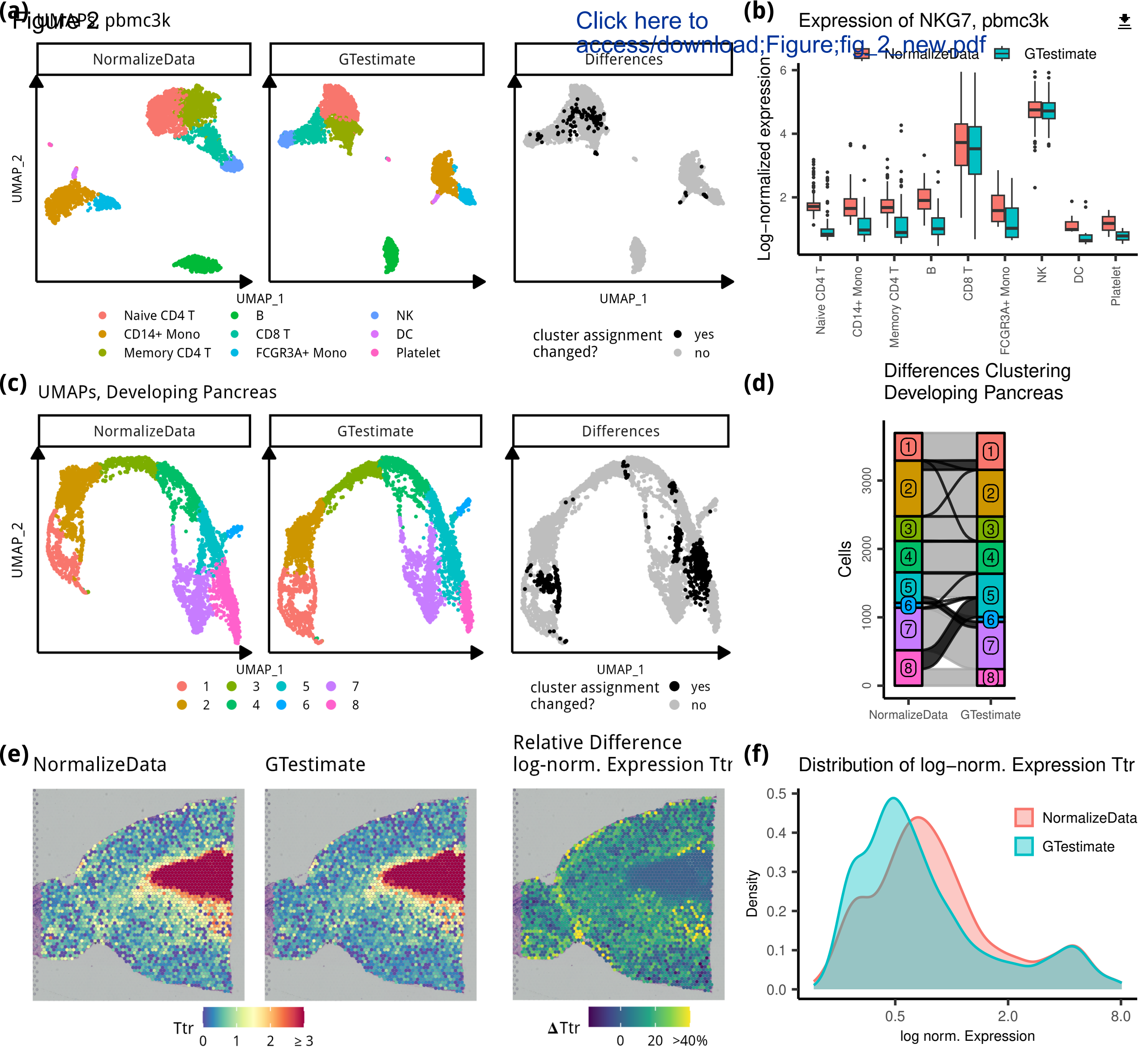

Figure 1

Click here to access/download;Figure;fig_1.pdf

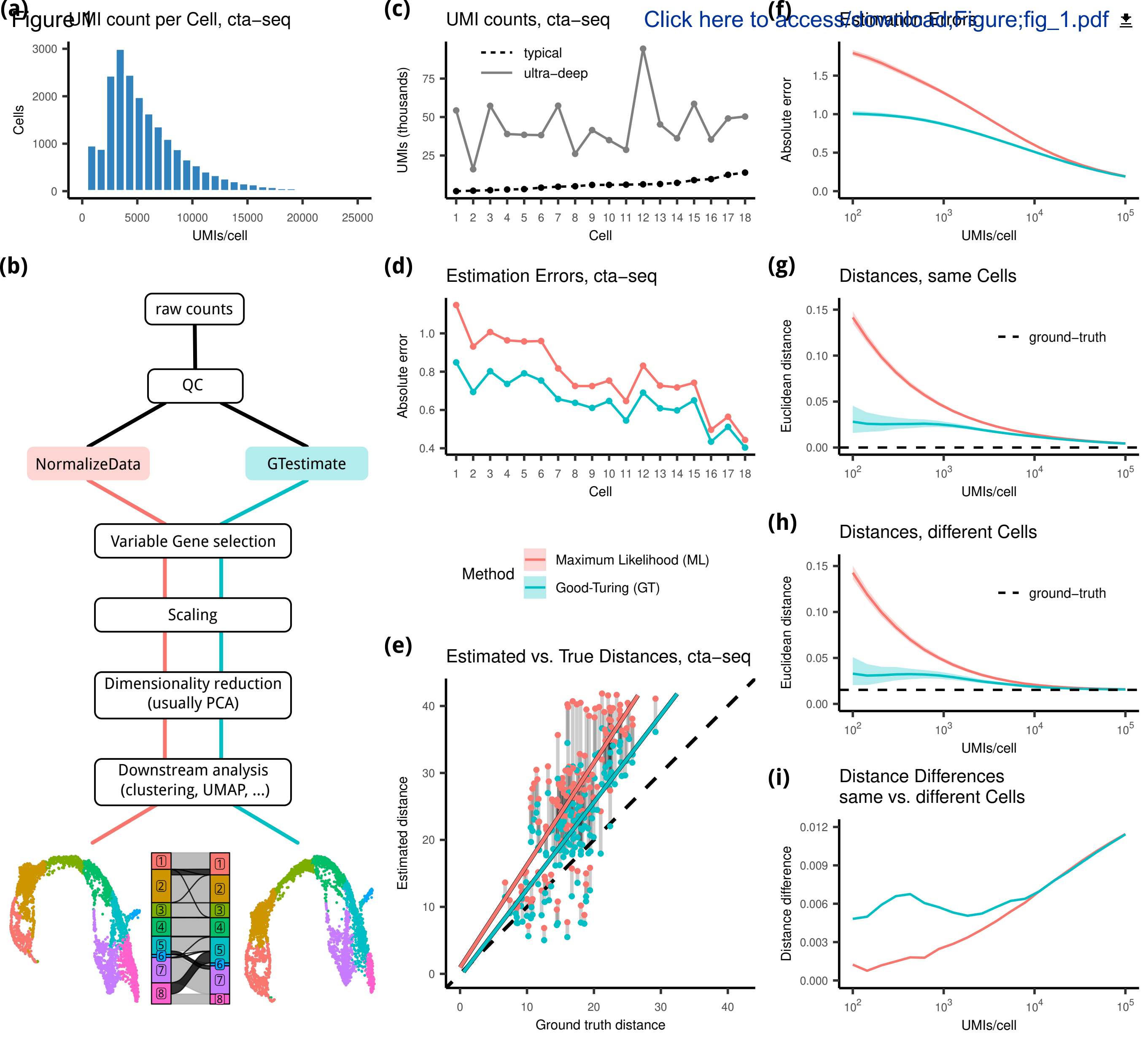

Supplementary Figure 9

Click here to access/download
Supplementary Material
ari_plot.pdf

Supplementary Figure 8

Click here to access/download
Supplementary Material
spatial_counts_supplementary_material.png

Supplementary Figure 6

Click here to access/download
Supplementary Material
spatial_clustering_supplementary_material.png

Supplementary Figure 3

Click here to access/download
Supplementary Material
feature_cta_seq_supplementary_material.pdf

Supplementary Figure 2

Click here to access/download
Supplementary Material
read_summary_supplementary_material.pdf

Supplementary Figure 4

Click here to access/download
Supplementary Material
more_markers_plot_supplementary_material.pdf

Supplementary Figure 1

Click here to access/download
Supplementary Material
gene_count_hist_supplementary_material.pdf

Supplementary Table 1 - Latex

Click here to access/download
Supplementary Material
pca_dists_supplementary_material.tex

Supplementary Figure 5

Click here to access/download
Supplementary Material
brain_umap_supplementary_material.png

Supplementary Figure 7

Click here to access/download
Supplementary Material
brain_jaccard_supplementary_material.pdf

Supplementary Figure 10

Click here to access/download
Supplementary Material
mm_cta_seq_supplementary_material.pdf

Supplementary Figure 11

Click here to access/download
Supplementary Material
mm_hist_supplementary_material.pdf
