# Supplementary material for: GTestimate: improving relative gene expression estimation in scRNA-seq using the Good–Turing estimator
Source: Gigascience. 2025 Oct 8;14:giaf084. doi: 10.1093/gigascience/giaf084 (PMC12569601; doi:10.1093/gigascience/giaf084)
Supplement: giaf084_Authors_Response_To_Reviewer_Comments_Revision_1 [file giaf084_authors_response_to_reviewer_comments_revision_1.pdf]

Revision-round	1
File-type	author-comment
Role	author
Name	Martin Fahrenberger
Date-type	author-comment-received
Date	25th March 2025

(GIGA)^n SCIENCE

Author's Response to Reviewer Comments

Summary:
We thank both reviewers for their valuable comments, which helped improve our manuscript. Based on their suggestions, we've made several key revisions:
Included additional comparisons with SCTransform through a new clustering analysis using an experimentally labeled dataset (Fig S9).
Clarified the Good-Turing estimator formula by explicitly including smoothing, addressing methodological transparency concerns.
Provided clearer justifications for methodological choices and corrected figure referencing issues.
These revisions have substantially enhanced the clarity and robustness of our manuscript.
As per Journal guidance we have registered GTestimate at bio.tools and SciCrunch.org (l. 360).
Detailed responses to Reviewer comments can be found below.

Reviewer 1:

We thank Reviewer 1 for their thoughtful and constructive comments, which have significantly improved our manuscript. Below, we address the comments point-by-point.

In this manuscript, Fahrenberger et al. propose a new scRNA-seq normalization method to more accurately report UMI counts of individual cells. They specifically use a Good-Turing estimator, compared with a more commonly used Maximum Likelihood estimator, to adjust raw UMI counts. Using their own cta-seq, a cell targeted PCR-amplification strategy, as ground truth, they compare their estimator with a traditional size-corrected estimator. Furthermore, they illustrate downstream changes using their method, including changes to clustering results and spatial transcriptomic readouts. The manuscript was a clear read and presents an interesting alternative solution to an often overlooked, but important, problem. However, there are some aspects of the manuscript that need to be addressed.

R1C1:
Some major content missing includes comparisons with more widely-used normalization methods throughout the manuscript, and better ground truth data sets

in their downstream analysis.

Author Response:
We appreciate this comment, however, we purposefully limited the comparisons to NormalizeData to keep all steps, other than the relative gene expression estimation, unchanged (Fig 1b). Our improved relative gene expression estimation is directly transferable to other methods such as scran's computeSumFactors or scanpy's normalize_total.

Direct comparisons to methods such as SCTransform, which involve additional data transformations, are only possible based on downstream results. Here, a lack of generally accepted ground-truth data-sets makes fair comparisons nearly impossible. One of the reasons why we designed cta-seq was to gain the best possible ground-truth data-set. We have now added to the main text (l. 104-109) to more clearly justify why methods such as e.g. SCTransform are not directly comparable.

We have also added clustering based analysis of a recent data-set which includes ground-truth cell-type annotations (l. 141-164 and Figure S9 and Methods l. 338-350). For this data-set we included SCTransform in our comparison. These results further highlight the strengths of GTestimate.

Specific comments are as follows:

R1C2:
l. 34: To my knowledge, most groups do not use a single division by total UMI count as the only normalization. Seurat has NormalizeData, but also heavily promotes scTransform, a completely different method. Many use log transform (as I believe was done here), some use quantile transform, others use regression techniques etc. It was odd to see these standard normalizations missing in comparisons. The authors should use such standard procedures to demonstrate the superiority of GT.

Author Response:
Many other scRNA-seq normalization methods apply additional transformations to the data or do not compute relative gene expression values. In the manuscript, we state that the calculation of relative gene expression with ML is inherent to all global-scaling methods (l. 33). Our comparison is specifically focused on improving the initial estimation for these methods, while keeping all other steps unchanged.

Regarding the usage frequency of different normalization methods: A look at recent scRNA-seq publications shows that "NormalizeData" and "SCTransform" are used roughly equally.
Additionally, Ahlmann-Eltze and Huber (2023) performed a large comparison of scRNA-seq data transformations/normalizations. They concluded that “delta methods” such as NormalizeData are as good as or better than other approaches, further justifying our focus on NormalizeData as a comparison.

R1C3:
l. 42: Is there a justification for the successor function being applied within the frequency ((cg + 1) / total) instead of outside ((cg / total) + 1) as is expected with

the Good-Turing estimation?

Author Response:
We have checked the formula again, it follows from equations 2 and 2' in Good (1953).

R1C4:
Furthermore, there is typically a smoothing function for erratic N_cg values, which I would expect with single-cell data. In the methods there is a brief mention of linear smoothing, but that would imply that the GT equation is
misleading and oversimplified. The actual equation should be included in the main text to avoid confusion.

Author Response:
We referred to our estimator as a Simple Good Turing estimator in the main text and cited [3] to indicate the use of the smoothing procedure.
To clarify, we now explicitly included the smoothing function S() in our main equation (GT) and added an additional reference to the smoothing procedure described by Gale and Sampson (1995) chapter 4 (l. 42-44). We believe this addition resolves potential confusion and enhances methodological transparency.

R1C5:
l. 58: Compared to 16,965 reads average per cell, what is the equivalent for the ultra-deep sequencing (not 23 million reads, as that is not 7.4 fold increase)?

Author Response:
The 7.4 fold increase is the ratio of, on average, 44,511 UMIs/cell (ultra-deep) to, on average, 6,048 UMIs/cell (typical). The 23 million reads/cell and the 16,965 reads/cell are the corresponding average numbers of raw reads per condition before UMI based deduplication.
This is now more clearly expressed (l. 59).

R1C6:
I am not entirely convinced on the use of cta-seq as a ground-truth for the cells, especially in comparison with ML. The authors should show that cta-seq has similar UMI and gene count distributions to more popular scRNA-seq technologies (e.g. 10x Chromium) or the application may be specific to cta-seq only.

Author Response:
We are uncertain which aspect of cta-seq Reviewer 1 is unconvinced by.

To clarify:
Our "typical" sample is a standard 10X Chromium data-set. We essentially used leftover material from the same 10X Chromium library prep for our "ultra-deep" sample. Fig 1a and Fig S1 show the UMI and gene count distribution of the "typical" sample, which is similar to other 10X Chromium data-sets.

The "ultra-deep" sample was generated through targeted amplification of the leftover material, using primers specific to the 18 selected cell-barcodes. The targeted PCR amplifications were performed in 18 separate reactions. Therefore, PCR-efficiency

biases, due to the different primers, would affect all cDNAs from the same cell equally. (We have adjusted the methods section to more clearly state the 18 separate PCR reactions. (l. 204-205))

This leaves potential differences in efficiency between the cells in our "ultra-deep" sample, these are present as we can see in Fig S2. However, they are mitigated through the UMI based deduplication of reads and by the fact that we only use relative gene expression levels in all our comparisons.

Assessment of the "ultra-deep" data based on gene-count or UMI count distributions is not possible due to the low number of only 18 cells.

R1C7:
l. 110: Instead of using unknown classification data sets, there are existing cell-sorted data sets with ground truths (many even on the 10x website). The authors should use these data sets to compare downstream analysis.

Author Response:
After some additional literature research we identified the liu data-set from Fu et al. 2024 as a good candidate for an experimentally labeled scRNA-seq data-set.

We used this data-set to compare GTestimate, NormalizeData and also SCTransform based on the Adjusted Rand Index (ARI) of the clustering results.
Our results show that GTestimate achieves the highest overall ARI at 0.874 compared to SCTransform's 0.822 and NormalizeData's 0.768.

We have added Figure S9 and lines 141-164 in the results section, as well as lines 338-350 in the Methods section.

R1C8:
l. 125: The spatial transcriptomic results were very subjective, with no statistical hypotheses.

Author Response:
The results in Fig 2e are mostly a proof-of-principle, highlighting an additional application for GTestimate.
Additionally, they highlight the differences in downstream results when using GTestimate instead of NormalizeData.

R1C9:
The entire manuscript is missing any sort of statistics when comparing methods, which is a major flaw and should be rectified.

Author Response:
As explained in R1C1 the lack of ground-truth prohibits the use of statistics to compare downstream results.
We specifically designed cta-seq to gain ground-truth relative gene expression values

and for this data the results are quite clear. Fig 1d, for example, shows that GT outperforms ML in 18 of 18 cells.

At the reviewer's request we added Fig S9, which now provides an additional comparison based on experimentally annotated cell-types.

Our results clearly indicate the superiority of GTestimate.

R1C10:
Here specifically, the color scale stops at 3, but does this carry over to the relative differential expression?

Author Response:
To compute the relative difference in gene expression we used the full range of log-normalized gene expression values.
For illustration purposes we only show the log-normalized gene expression difference between 0 and 3 to highlight the difference for the lowly expressed spots, which are mostly affected by the switch from ML to GT.

R1C11:
The claim is that it is constant, but if they are all greater than 3 then they must be quite variable, so it is surprising to see such a constant value of 0. Maybe the complete color scale should be shown on all figures to clarify this.

Author Response:
The choroid plexus (red area) is characterized by high expression values, in such situations the relative difference between NormalizedData and GTestimate is small (as illustrated in Fig 2f), thus, it is not surprising that the differences all appear to be close to zero.

R1C12:
From my understanding of the manuscript, the 18 cells for analysis and comparison were chosen based on a typical Seurat analysis. This technique introduces a range of biases into the comparison and makes the argument a bit circular.

For a bias example, the top 2000 most variable genes were used, suggesting that entire classes of genes may be ignored even when highly or lowly expressed, such as housekeeping genes.

There also appears to be many steps that were not entire justified outside of a "typical analysis", for example excluding a cluster in the analysis (just because it was not that large?), only selection 18 cells (why 6 from each cluster?), removing cells with less than 1000 expressed genes or over 8% mitochrondrial reads (this may be an issue, and removing specific cell types or proliferating cells, this should be a bivariate removal with justification). All of these filterings remove generalizeability of GT.

Author Response:
We appreciate the reviewer raising this important concern. To clarify, the choice of

cells used for benchmarking GT versus ML was intended purely to ensure diversity among the selected cells, rather than to achieve optimal results for any particular normalization method. In fact, the exact selection of these 18 cells (e.g., choosing 6 cells per cluster or excluding small clusters) is relatively unimportant; selecting 18 cells at random would also have been a valid approach. The key purpose was to include a diverse collection of cell types with differing transcriptional profiles, spanning both low quality and high quality cells. This ensures robustness of our conclusions across both biological and technical variance.

We chose 18 cells as this seemed to be a large enough set to draw conclusions without diluting our sequencing efforts among too many cells.

Regarding the filtering steps applied (e.g., exclusion of cells with fewer than 1000 expressed genes or those exceeding 8% mitochondrial reads), we followed common quality-control practices frequently used in standard scRNA-seq analyses (https://satijalab.org/seurat/articles/pbmc3k_tutorial). Our intention was to adopt a transparent, standard workflow that readers can easily replicate.

The generalizability of GTestimate is not impacted by the data-set chosen, since GTestimate was not used during cell selection for cta-seq, and cell selection was not optimized to favor GTestimate.

R1C13:
Supplementary Figures in the text hyperlink to the main figures which is confusing. More importantly, the caption of Supplementary Figures read "Figure" rather than "Supplementary Figures".

Author Response:
This has been corrected in the revised manuscript.

Reviewer 2:

We thank Reviewer 2 for the comments. We hope our point-by-point response alleviates Reviewer 2's concerns.

This paper introduces a Good-Turing (GT) estimation scheme for relative gene expression estimation and cell-cell distance estimation. The proposed methods, namely GTestimate, claims to improve upon conventional normalization methods by accounting for unobserved genes.
The idea behind this contribution is fairly straightforward - since the relative gene expression is of large alphabet, a GT estimator is expected to preform better than a naive ML approach. However, I am not convinced that the authors applied it correctly.

R2C1:
First, the proposed GT estimator (as appears in (GT)) in the text), assigns a zero estimate to unobserved genes ($C_g = 0$). This contradicts the entire essence of using a

GT estimator.

Author Response:
Our decision to assign zero estimates to unobserved genes (where C_g = 0) was deliberate and justified by biological considerations and does not contradict the essence of Good-Turing estimation.
Count-matrices in scRNA-seq typically contain 20,000-30,000 genes to accommodate all expressed genes across the different cell-types. However, current understanding of biology tells us that any individual cell type expresses only a subset of these genes.
This creates a scenario where:
The true alphabet size (number of genuinely expressed genes per cell type) is unknown and varies between cell types
We perform thousands of individual GT estimations (one per cell)
There is no reliable way to determine which specific unobserved genes should receive portions of the missing mass in the current cell
Gale and Sampson (1995) [3] acknowledged that GT estimators can not by themselves redistribute the missing mass in §9 of their paper: "Good-Turing techniques give an overall estimate P_0 for the probability of all unseen species taken together, but in themselves they can give no guide to the individual probabilities of the separate unseen species."
They further note: "Provided the number of unseen species is known, the obvious approach is to divide P_0 equally between the species. But this is a very unsatisfactory technique."
They then go on to discuss a data-set specific approach to the problem, which can not be transferred to scRNA-seq data-sets. Developing a suitable way to redistribute the missing mass in scRNA-seq data-sets remains an open problem which may warrant further research.

We also did not want to assign small non-zero expression values to all unobserved genes, as this may introduce unpredictable effects across the various downstream analysis tools and violate their assumptions.

R2C2:
Second, it makes no since to use this expression for every Cg > 0. In fact, any reasonable GT based estimator applies GT for relatively small Cg, and ML estimator for large Cg. See [1] for a through discussion. The choice of a threshold between "small" and "large" Cg's is subject to many studied (for example [2], [1]), but it makes no sense to use the above expression for any Cg.

Author Response:
This comment is a bit surprising to us, since many of the seminal papers on GT estimators suggested using the same GT expression independent of C_g.

After this remark we reexamined the methods introduced in [1] and [2].
The two papers introduce a combined ML and GT estimator with a switching-rule to decide which estimate is used. Drukh et al. suggest using the GT estimate for any C_g <= N^(2/5) and the ML estimate for C_g > N^(2/5), where N is the sample size.
However, we remain unconvinced of the usefulness of such an estimator for our data-

sets for two reasons:

First, there are only very few genes per cell for which this switching rule comes into effect. When tested on the pbmc3k data-set (also used in the paper), a quick analysis revealed that, on average, the switching rule would use ML instead of GT for 13.34 genes per cell out of the, on average, 849 expressed genes.

Second, by definition this rule would only apply for the highest C_g values while, at the same time, the relative difference between the ML estimates and GT estimates gets smaller for higher values C_g. This indicates that a switch between the two methods will only have marginal effects. For the pbmc3k data-set the affected genes, on average, showed an absolute relative difference between the GT and the ML estimate of 0.024, confirming that a combined estimator would only make small adjustments.

R2C3:
Finally, notice that if $N_{Cg} > 0$ for some g but $N_{Cg+1} = 0$, the proposed estimator is not defined. There exists several smoothing solutions for such cases (for example [3]), but they need to be properly discussed.

Author Response:
We referred to our estimator as a Simple Good Turing estimator in the main text and cited [3]. We also discussed the need for smoothing of the N_{C_g} values in the methods section (l. 183-187).
To further clarify, we now explicitly included the smoothing function S() within our main equation (GT) and added an additional reference to the smoothing procedure described by [3] (l. 42-44). We believe this addition resolves potential confusion and enhances methodological transparency.

R2C4:
to conclude, I am not sure what is the effect of these issues on the experiments in the paper, which makes it difficult to assess the results.

Author Response:
We hope that our point-by-point responses above lay any concerns to rest and that the results in our paper now speak for themselves.

REFERENCES

[1] A. Painsky, "Convergence guarantees for the good-turing estimator," Journal of Machine Learning Research,
vol. 23, no. 279, pp. 1-37, 2022.
[2] E. Drukh and Y. Mansour, "Concentration bounds for unigram language models." Journal of Machine Learning
Research, vol. 6, no. 8, 2005.
[3] W. A. Gale and G. Sampson, "Good-Turing frequency estimation without tears," Journal of quantitative
linguistics, vol. 2, no. 3, pp. 217-237, 1995.
