# Supplementary material for: GTestimate: improving relative gene expression estimation in scRNA-seq using the Good–Turing estimator
Source: Gigascience. 2025 Oct 8;14:giaf084. doi: 10.1093/gigascience/giaf084 (PMC12569601; doi:10.1093/gigascience/giaf084)
Supplement: giaf084_Authors_Response_To_Reviewer_Comments_Revision_2 [file giaf084_authors_response_to_reviewer_comments_revision_2.pdf]

Revision-round	2
File-type	author-comment
Role	author
Name	Martin Fahrenberger
Date-type	author-comment-received
Date	26th May 2025

(GIGA)^n SCIENCE

Author's Response to Reviewer Comments

Summary:
We thank the reviewer for the valuable feedback. However we remain convinced that the comparative analyses presented in the manuscript, including the newly added evaluation on the Liu data-set (Fig. S9), are comprehensive and unbiased. Below we argue why the comparisons made in our manuscript are adequate.
On a side note, the accompanying data-set is now accessible via controlled access at the European Genome-Phenome Archive (EGA) via accession number EGAD50000001338 (l. 367).

Point-by-point Response:

R1C1:
I thank the authors for their additional experiments comparing other normalization methods with GTestimate. However, there are still some results I am unclear on that are not fully explained by the response.

Author Response:
We hope that our individual responses below convince the reviewer and alleviate any remaining concerns.

R1C2:
I am still confused why there is a lack of any type of statistics for statements about, for example, improvements to downstream results. The authors state that "the lack of ground-truth prohibits the use of statistics" but the statistical test is unrelated to ground truth, rather the comparisons between methods. This ensures that the results are not spurious findings.

Author Response:
When statistically comparing two methods, the result only indicates whether the two methods provide significantly different results or not. The test result makes no statement as to which result is the 'correct' one. The test does not help to present one method or the other as superior. Moreover, we would like to emphasize:

- Visual differences in Fig 2 are already clear. The differences between methods in Fig 2 are already pronounced and easy to interpret.

- Significance does not imply utility. A method can yield a clustering which is significantly different from other clusterings, yet biologically uninformative. Without ground-truth labels, such tests only show statistical distinctness, not biological relevance.
- Results in Figure 1 are very clear. For the ML vs. GT comparison in Fig. 1 (where ground-truth expression values are available) the superiority of GTestimate is already apparent from the presented data and visualization and supported by the underlying theory on Good-Turing estimators. Therefore additional tests are not necessary.

R1C3:
Figure S9 attempts to compare several normalizations which is greatly appreciated, but was done in a biased manner. The authors potentially introduce overfitting by using a range of resolution values rather than the default (1), then use the maximum ARI to determine performance.

Author Response:
We are not sure what is meant by overfitting in this context. It is standard practice in statistics to adjust a parameter (here resolution) to obtain a better fit to the ground truth and to better understand the influence of the parameter. We, like many others, follow this strategy.

R1C4:
However, in a real-life scenario ARI is unknown with no ground truth, so would mostly be defined at a resolution of 1, unless the users want to see more clusters unrelated to the unknown ARI.

Author Response:
It is true that the ARI is not available in a regular scRNA-seq study, however we would still expect researchers to optimize the resolution parameter until they are satisfied with the clustering results based on their domain-knowledge.

For example the popular online book “Single-cell best practices” recommends trying multiple resolution settings (https://www.sc-best-practices.org/cellular_structure/clustering.html) and choosing based on the desired outcome. This desired outcome may, for example, be a certain number of clusters, or clear cell-type identities.

In fact a resolution of 1 would result in overclustering of the Liu data-set for all three tested normalization methods. While the ground-truth annotates 10 different cell-types, normalization with NormalizeData, GTestimate and SCTransform, followed by Louivan clustering at a resolution of 1 resulted in 12, 15 and 16 clusters respectively.

R1C5:
As such, the authors are ignoring all of the resolution values where GTestimate performs worse than, say, SCTransform.

Author Response:
While It is true that GTestimate performs slightly worse than SCTransform at a resolution of 1 (for this data-set), we strongly disagree with this statement. Figure S9

shows the ARI for all 15 resolutions. Figure S9 also shows that GTestimate outperforms NormalizeData at 14 of 15 resolutions and outperforms SCTransform at 10 of 15 resolutions, we state this clearly in l. 161-162.

R1C6:
As such, this analysis is problematic. To resolve this issue, I suggest using an alternative approach, such as setting the number of clusters with an alternate method such as k-means clustering or spectral clustering to the number of known cell types in the sample.

Author Response:
While this is a creative suggestion, we are convinced that our current analysis of this data-set is better suited in the context of this manuscript, because:

1. Louivan clustering is state-of-the-art. Our goal here is to show the performance of GTestimate while using the most common methods, i.e. Louivan clustering; the default currently used by both Seurat and scanpy.
2. Alternative clustering methods are beyond the scope. Adding alternative clustering methods would increase the complexity of the manuscript considerably and would potentially introduce its own biases and make the conclusions harder to interpret for the end-user, who will most likely be using Louvian clustering.
3. Best clustering results are not achieved at n=10. Using ARI as a measure of clustering performance (and the Louivan algorithm for clustering) our analysis shows that the best representation of the ground-truth clustering is not achieved at 10 clusters. Across our 15 resolutions from 0.1 to 1.5 we observed that:
- NormalizeData achieves its best ARI (0.768) at a resolution of 0.7 with 11 clusters.
- GTestimate achieves its best ARI (0.874) at a resolution of 0.6 with 12 clusters.
- SCTransform achieves its best ARI (0.822) at a resolution of 0.4 with 9 clusters.

While the observed ARI-values show that none of these clusterings are perfect, optimizing the clustering further is not the goal of this manuscript. We did however show that GTestimate enables the best clustering results in this setup.

Conclusion:
1. Additional statistical tests for downstream results can only show a difference in results, not a difference in quality. This difference is already clear in our Figures.
2. The resolution parameter is routinely adjusted in scRNA-seq data analysis and therefore the results have to be examined at a wide range of possible settings.
3. Louivan clustering is state-of-the-art, and should therefore be used for benchmarking.
4. In our analysis GTestimate outperforms NormalizeData and SCTransform by all measures we looked at: mean ARI, max ARI and 'number of wins' i.e. number of resolutions at which a method is the best choice.
