# Supplementary material for: GTestimate: improving relative gene expression estimation in scRNA-seq using the Good–Turing estimator
Source: Gigascience. 2025 Oct 8;14:giaf084. doi: 10.1093/gigascience/giaf084 (PMC12569601; doi:10.1093/gigascience/giaf084)
Supplement: giaf084_Reviewer_1_Report_Original_Submission — Dr. Gregory Schwartz -- 9/30/2024 [file giaf084_reviewer_1_report_original_submission.pdf]

Revision-round	0
File-type	reviewer-report
Role	reviewer 1
Name	Dr. Gregory Schwartz
Date-type	reviewer-report-received
Date	30th September 2024

Reviewer Report

Title: GTestimate: Improving relative gene expression estimation in scRNA-seq using the Good-Turing estimator

Version: Original Submission Date: 9/30/2024

Reviewer name: Dr. Gregory Schwartz

Reviewer Comments to Author:

In this manuscript, Fahrenberger et al. propose a new scRNA-seq normalization method to more accurately report UMI counts of individual cells. They specifically use a Good-Turing estimator, compared with a more commonly used Maximum Likelihood estimator, to adjust raw UMI counts. Using their own cta-seq, a cell targeted PCR-amplification strategy, as ground truth, they compare their estimator with a traditional size-corrected estimator. Furthermore, they illustrate downstream changes using their method, including changes to clustering results and spatial transcriptomic readouts. The manuscript was a clear read and presents an interesting alternative solution to an often overlooked, but important, problem. However, there are some aspects of the manuscript that need to be addressed. Some major content missing includes comparisons with more widely-used normalization methods throughout the manuscript, and better ground truth data sets in their downstream analysis. Specific comments are as follows: l. 34: To my knowledge, most groups do not use a single division by total UMI count as the only

normalization. Seurat has NormalizeData, but also heavily promotes scTransform, a completely different method. Many use log transform (as I believe was done here), some use quantile transform, others use regression techniques etc. It was odd to see these standard normalizations missing in comparisons. The authors should use such standard procedures to demonstrate the superiority of GT. l. 42: Is there a justification for the successor function being applied within the frequency ((cg + 1) / total) instead of outside ((cg / total) + 1) as is expected with the Good-Turing estimation? Furthermore, there is typically a smoothing function for erratic N_cg values, which I would expect with single-cell data. In the methods there is a brief mention of linear smoothing, but that would imply that the GT equation is misleading and oversimplified. The actual equation should be included in the main text to avoid confusion. l. 58: Compared to 16,965 reads average per cell, what is the equivalent for the ultra-deep sequencing (not 23 million reads, as that is not 7.4 fold increase)? I am not entirely convinced on the use of cta-seq as a ground-truth for the cells, especially in comparison with ML. The authors should

show that cta-seq has similar UMI and gene count distributions to more popular scRNA-seq technologies (e.g. 10x Chromium) or the application may be specific to cta-seq only. l. 110: Instead of using unknown classification data sets, there are existing cell-sorted data sets with ground truths (many even on the 10x website). The authors should use these data sets to compare downstream analysis. l. 125: The spatial transcriptomic results were very subjective, with no statistical hypotheses. The entire manuscript is missing any sort of statistics when comparing methods, which is a major flaw and should be rectified. Here specifically, the color scale stops at 3, but does this carry over to the relative differential expression? The claim is that it is constant, but if they are all greater than 3 then they must be quite variable, so it is surprising to see such a constant value of 0. Maybe the complete color scale should be shown on all figures to clarify this. From my understanding of the manuscript, the 18 cells for analysis and comparison were chosen based on a typical Seurat analysis. This technique introduces a range of biases into the comparison and makes the argument a bit circular. For a bias example, the top 2000 most variable genes were used, suggesting that entire classes of genes may be ignored even when highly or lowly expressed, such as housekeeping genes. There also appears to be many steps that were not entire justified outside of a "typical analysis", for example

excluding a cluster in the analysis (just because it was not that large?), only selection 18 cells (why 6 from each cluster?), removing cells with less than 1000 expressed genes or over 8% mitochrondrial reads (this may be an issue, and removing specific cell types or proliferating cells, this should be a bivariate removal with justification). All of these filterings remove generalize ability of GT. Supplementary Figures in the text hyperlink to the main figures which is confusing. More importantly, the caption of Supplementary Figures read "Figure" rather than "Supplementary Figures".
