# Supplementary material for: GTestimate: improving relative gene expression estimation in scRNA-seq using the Good–Turing estimator
Source: Gigascience. 2025 Oct 8;14:giaf084. doi: 10.1093/gigascience/giaf084 (PMC12569601; doi:10.1093/gigascience/giaf084)
Supplement: giaf084_Reviewer_1_Report_Revision_1 — Dr. Gregory Schwartz -- 3/27/2025 [file giaf084_reviewer_1_report_revision_1.pdf]

Revision-round	1
File-type	reviewer-report
Role	reviewer 1
Name	Dr. Gregory Schwartz
Date-type	reviewer-report-received
Date	27th March 2025

Reviewer Report

Title: GTestimate: Improving relative gene expression estimation in scRNA-seq using the Good-Turing estimator

Version: Revision 1 **Date: 3/27/2025**

Reviewer name: Dr. Gregory Schwartz

Reviewer Comments to Author:

I thank the authors for their additional experiments comparing other normalization methods with GTestimate. However, there are still some results I am unclear on that are not fully explained by the response. I am still confused why there is a lack of any type of statistics for statements about, for example, improvements to downstream results. The authors state that "the lack of ground-truth prohibits the use of statistics" but the statistical test is unrelated to ground truth, rather the comparisons between methods. This ensures that the results are not spurious findings. Figure S9 attempts to compare several normalizations which is greatly appreciated, but was done in a biased manner. The authors potentially introduce overfitting by using a range of resolution values rather than the default (1), then use the maximum ARI to determine performance. However, in a real-life scenario ARI is unknown with no ground truth, so would mostly be defined at a resolution of 1, unless the users want to see more clusters unrelated to the unknown ARI. As such, the authors are ignoring all of the resolution values where GTestimate performs worse than, say, SCTransform. As such, this analysis is problematic. To resolve this issue, I suggest using an alternative approach, such as setting the number of clusters with an alternate method such as k-means clustering or spectral clustering to the number of known cell types in the sample.
