# Supplementary material for: GTestimate: improving relative gene expression estimation in scRNA-seq using the Good–Turing estimator
Source: Gigascience. 2025 Oct 8;14:giaf084. doi: 10.1093/gigascience/giaf084 (PMC12569601; doi:10.1093/gigascience/giaf084)
Supplement: giaf084_Reviewer_2_Report_Original_Submission — Dr. Amichai Painsky -- 12/15/2024 [file giaf084_reviewer_2_report_original_submission.pdf]

Revision-round	0
File-type	reviewer-report
Role	reviewer 2
Name	Dr. Amichai Painsky
Date-type	reviewer-report-received
Date	15th December 2024

Reviewer Report

Title: GTestimate: Improving relative gene expression estimation in scRNA-seq using the Good-Turing estimator

Version: Original Submission **Date: 12/15/2024**

Reviewer name: Dr. Amichai Painsky

Reviewer Comments to Author:

This paper introduces a Good-Turing (GT) estimation scheme for relative gene expression estimation and cell-cell distance estimation. The proposed methods, namely GTestimate, claims to improve upon conventional normalization methods by accounting for unobserved genes. The idea behind this contribution is fairly straightforward - since the relative gene expression is of large alphabet, a GT estimator is expected to preform better than a naive ML approach. However, I am not convinced that the authors applied it correctly. First, the proposed GT estimator (as appears in (GT)) in the text), assigns a zero estimate to unobserved genes (Cg = 0). This contradicts the entire essence of using a GT estimator. Second, it makes no since to use this expression for every Cg > 0. In fact, any reasonable GT based estimator applies GT for relatively small Cg, and ML estimator for large Cg. See [1] for a through discussion. The choice of a threshold between "small" and "large" Cg's is subject to many studied (for example [2], [1]), but it makes no sense to use the above expression for any Cg. Finally, notice that if N_{Cg} > 0 for some g but N_{Cg+1} = 0, the proposed estimator is not defined. There exists several smoothing solutions for such cases (for example [3]), but they need to be properly discussed. to conclude, I am not sure what is the effect of these issues on the experiments in the paper, which makes it difficult to assess the results. REFERENCES [1] A. Painsky, "Convergence guarantees for the good-turing estimator," Journal of Machine Learning Research, vol. 23, no. 279, pp. 1-37, 2022. [2] E. Drukh and Y. Mansour, "Concentration bounds for unigram language models." Journal of Machine Learning Research, vol. 6, no. 8, 2005. [3] W. A. Gale and G. Sampson, "Good-Turing frequency estimation without tears," Journal of quantitative linguistics, vol. 2, no. 3, pp. 217-237, 1995.
